# Elucidation of resistance signaling and identification of powdery mildew resistant mapping loci (*ClaPMR2*) during watermelon-*Podosphaera xanthii* interaction using RNA-Seq and whole-genome resequencing approach

**DOI:** 10.1038/s41598-020-70932-z

**Published:** 2020-08-20

**Authors:** Mihir Kumar Mandal, Haktan Suren, Chandrasekar Kousik

**Affiliations:** 1 U.S. Vegetable Laboratory, USDA, ARS, 2700 Savannah Highway, Charleston, SC 29414 USA; 2 Department of Biology, Claflin University, Orangeburg, SC 29115 USA; 3 ORISE Participant Sponsored by the U.S. Vegetable Laboratory, USDA, ARS, 2700 Savannah Highway, Charleston, SC 29414 USA; 4 Department of Forest Resources and Environmental Conservation, Virginia Tech, Blacksburg, VA 24061 USA

**Keywords:** Plant immunity, Comparative genomics

## Abstract

Watermelon is an important vegetable crop and is widely cultivated in USA with an approximate global production of > 100 million tons. Powdery mildew (PM) caused by *Podosphaera xanthii* is a major production-limiting factor on watermelon and other cucurbits. Numerous PM and multiple disease resistant (MDR) watermelon germplasm lines have been developed by the USDA in Charleston, SC. To gain a better understanding of the innate and activated molecular defense mechanisms involved during compatible and incompatible PM-watermelon interactions, we inoculated PM susceptible (USVL677-PMS) and resistant (USVL531-MDR) watermelon plants with 10^5^ conidia ml^−1^ of *P. xanthii*. RNA-seq profiling was done on leaf samples collected at 0, 1, 3, and 8 days post inoculation (DPI). A total of 2,566 unique differentially expressed genes (DEGs) were identified between compatible and incompatible interactions with *P. xanthii*. The compatible interactions resulted in distinct plant gene activation (> twofold unique transcripts, 335:191:1762 :: 1:3:8 DPI) as compared to incompatible interaction (> twofold unique transcripts, 314:681:487 :: 1:3:8 DPI). Further, comparative whole-genome resequencing analysis of USVL531-PMR, USVL677-PMS and four introgressed PM resistant recombinant inbred lines (RIL, USVL531-PMR × USVL677-PMS) were performed to identify the region of PM resistance introgressed break points along with other traits inherent by USVL531-PMR by comparing the SNPs and InDels. Based on SNPs identification and CAPS markers, the resistance gene was identified as *ClaPMR2, Citrullus lanatus PM Resistance gene 2* {Chr2 : 26750001 .. 26753327 (−)}*,* a NBS-LRR resistance protein (R) with homology to the *Arabidopsis thaliana* PM resistance protein, RPW8. The transcriptome data also revealed a complex regulatory network associated with the introgressed junctions mediated by PM resistance R proteins (R genes) that may involve multiple signal regulators and transducers, carbohydrate metabolism, cell wall modifications and the hormone-signaling pathway.

## Introduction

**Figure 1:**
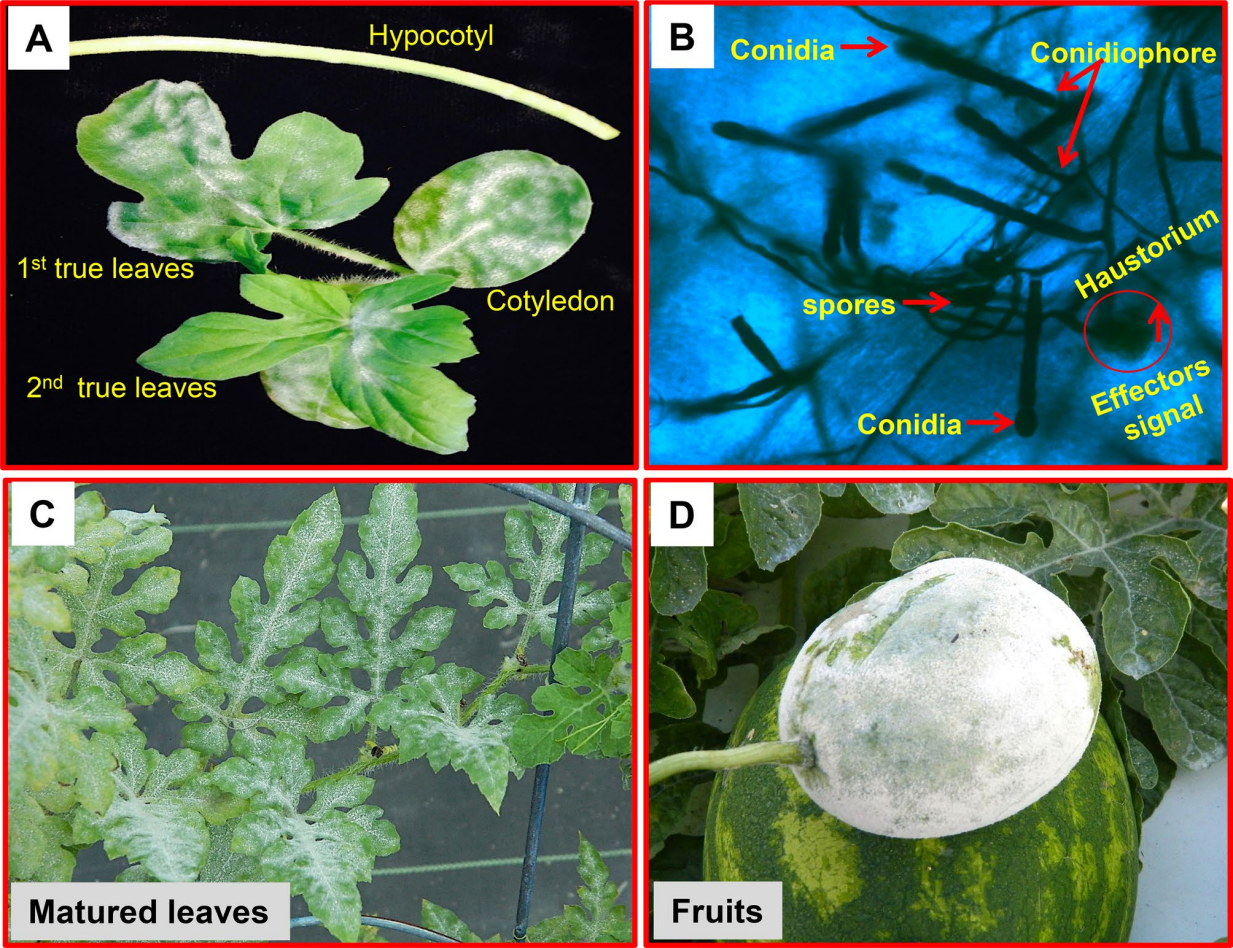
Symptoms of powdery mildew (PM) on watermelon caused by the pathogen *Podosphaera xanthii.* (**A**) PM conidial development on leaves and stem of 3–4 week old watermelon plant; (**B**) Trypan-blue stained *P. xanthii* hyphae collected from watermelon lines (USVL677-PMS) at 8 DPI with powdery mildew fungus; (**C**) PM conidial development in 7–8 week old matured leaves; (**D**) PM conidial development in fruit.

Powdery mildew (PM) caused by *Podosphaera xanthii* (formerly *Sphaerotheca fuliginea*) (Perez Garcia; Fukino) is a major foliar disease affecting greenhouse and field-grown watermelon and other cucurbit crops throughout the world^1–3^. The disease is considered a major limiting factor in cucurbit production and causes significant yield loss in United States of America (USA)^1,4–6^. The obligate biotrophic pathogen, *P. xanthii* is characterized by appearance of white powdery symptoms on above ground plant parts consisting of mycelium, conidiophores and conidia. It can infect all developmental stages of the crop including hypocotyls, cotyledons, stem, leaves and fruit (Fig. 1)^1–3,6–11^. Infection at early stage of the plant by *P. xanthii* reduces seedling vigor, causes premature desiccation of leaves, and predisposes them to other secondary pathogens^11,15^. Fruit infection is also known to reduce yield and marketable quality^12^. In recent years PM has been reported to occur more frequently on watermelon and other cucurbits across different parts of USA^4,12–18^. This has prompted use of synthetic fungicides to manage PM of watermelon^4,18,19^. However, excessive and long-term use of fungicides is not considered environmentally favorable due to its residual toxicity and increased selection pressure on PM pathogen populations leading to increased resistance to the fungicides^20^ and therefore, alternative management strategies are needed^21^.

In recent years, breeding for PM resistance by utilizing available resistant PI (plant introductions)/ germplasms has been achieved^2^. However, these watermelon varieties either lack the red-fleshed trait or the high brix (sugar) contents, which makes them difficult to release as commercial cultivars. In addition, selection for simultaneous inheritance of both traits (sugar/red-fleshed and PM resistance) during breeding program is a tedious process and can take several years. The presence of diverse races of the cucurbit PM pathogen *P. xanthii* further limits the production of watermelon and other cucurbits across the US states and globally^1,2,12–14,22^. With high PM pressure during growing season, development of new resistant germplasms with broad resistance to diverse PM races is a laborious and time consuming process that requires extensive selection of PI (plant introduction with resistant trait) lines and the utilization conventional breeding approaches to develop commercial quality lines with PM resistance.

A previous genetic inheritance study mapped PM resistance by utilizing a F2 population developed from a cross between watermelon variety Arka Manik (AM) and TS34 (TS), which led to the identification of a quantitative trait loci (QTL) region (*pmr2.1*) associated with PM resistance^23^. Though QTL can provide a genetic basis for PM resistance, knowledge of the specific genes directly involved in PM resistance as well as the molecular defense mechanisms involved in such PM-watermelon interaction is lacking. To our knowledge, a comprehensive trancriptomic study to identify DEGs during PM inoculation has not been previously conducted in watermelon. Considering the complex biotrophic nature of the PM pathogen and with the availability of the recent draft sequence of the watermelon genome^24^ (www.icugi.org/pub/genome/watermelon/97103/v1/), we hypothesized that a better understanding of the novel transcriptional regulatory mechanisms involved in the watermelon-*P. xanthii* interaction will lead to identification of key genes involved in host defense, from initial PM priming (spore germination/ pathogen recognition) to late colonization of the fungus. This will help advance our knowledge of the role of diverse biological pathways and/or molecular mechanisms associated with PM resistance and susceptibility in watermelon. In order to support our hypothesis, we conducted a time-course comparative transcriptome analysis, utilizing RNA-seq technology on PM susceptible watermelon leaves USVL677-PMS (PMS: PM susceptible), and on a multi-disease resistant line, USVL531-MDR (MDR: multi-disease resistant; PM resistant) infected with the PM pathogen *P. xanthii*. Further, comparative whole-genome resequencing analysis of parent line: USVL531-MDR, USVL677-PMS and four introgressed PM resistant F_5_ RIL lines derived from a cross of USVL531-MDR × USVL677-PMS were performed to identify the region of PM resistant introgressed break points along with other traits inherent by the multi-disease resistant line USVL531-MDR by comparing the SNPs and InDels associated with it.

## Materials and methods

### Plant materials and growth conditions

Seeds of watermelon germplasm lines USVL531-MDR (S_5_ derived from PI 494531) and USVL677-PMS (derived from PI 269677) were seeded in sterilized Metro-Mix soil (Sun Gro Horticulture, Bellevue, WA) in 50 cell trays. USVL677-PMS is susceptible to *P. xanthii* and *P. capsici,* whereas USVL531-MDR is resistant to both pathogens^3,21^. The trays were kept on a heated mat at 37 °C for 3–4 days for uniform seed germination. Seedlings with emerging cotyledons were kept in a room at 25 °C and 60–65% RH with 16 h photoperiod provided by cool white fluorescent bulbs with light intensity of 120 μmol m^−2^ s^−1^. Soil was fertilized once using Scotts Peter’s 20:10:20 peat lite special general fertilizer that contained 8.1% ammonical nitrogen and 11.9% nitrate nitrogen. Plants were irrigated using deionized or tap water.

### Powdery mildew isolate and PM leaf inoculation

For leaf inoculations, 4-week old watermelon seedlings (USVL531-MDR, USVL677-PMS) having 1–2 fully expanded true leaves were selected. A local isolate of the PM pathogen, *P. xanthii,* B108ML^1,12–14^ collected from watermelon plants in the greenhouse and routinely maintained in a growth chamber on ‘Early Prolific Straight Neck (EPSN)’ squash plants was used for inoculation. The watermelon seedlings maintained in a growth chamber (Temp 25 °C, RH 60–65%, photoperiod 16 h, light intensity 120 μmol m^−2^ s^−1^) were sprayed with a conidial suspension (10^5^ condia ml^−1^ in 0.02% tween 20) as described before^1,2,12–14,21^. Three separate plants of each genotype (USVL531-MDR, USVL677-PMS) were sampled for paired-end RNA-seq library preparation at four different time-points; 0 h (un-inoculated control), 24 h [1 Day post inoculation (DPI)], 72 h (3 DPI) and 192 h (8 DPI) with *P. xanthii*. The leaf samples collected from different plants with or without pathogen inoculation were directly frozen in liquid nitrogen and then stored at − 80 °C until RNA extraction.

### Trypan blue staining and light microscopic observation of PM pathogen

Staining of fungal mycelium on PM infected leaf samples was carried out as reported earlier^21^. In summary, the PM infected leaves were cut into small discs and kept in 12-well plates containing 2.5 mL of clearing solution (acetic acid:ethanol :: 1:3, v/v). The plates were sealed and placed on a rotary shaker set at 100 rpm for 12 h. The clearing solution was removed and replaced with 2 mL of a second clearing solution (acetic acid:ethanol:glycerol :: 1:5:1, v/v/v). The plates were then sealed and placed on a rotary shaker (100 rpm) for 3 h. The second clearing solution was removed and replaced with the trypan-blue staining solution (0.01% trypan-blue in lactoglycerol). The plates were then placed on a rotatry shaker (100 rpm) overnight. The staining solution was discarded and leaf samples were rinsed with 60% glycerol (3 ×) and mounted on a clean glass slide and covered with a cover slip. Samples mounted on glass slides were observed with a Leica compound microscope (Leica Microsystems Inc. IL, USA https://www.leica-microsystems.com/home) with attached Lumenera Infinity 3 CCD camera attached to the microscope.

### Total RNA extraction, RNA-seq library preparation and Illumina sequencing

Total RNA was isolated from frozen true leaf tissues using TRIzol reagent (Invitrogen, Carlsbad, CA, USA) following manufacturer’s instructions. RNA purification steps and on-column DNase digestion was performed using the QIAGEN RNeasy Mini Kit as suggested by the manufacturer’s instructions (QIAGEN, Hilden, Germany). The total RNA qualities were further analyzed using Agilent RNA 6000 Nano Kit (Agilent Technologies Inc., Santa Clara, CA, USA) ran on a Agilent 2100 Bioanalyzer, with average RNA RIN values > 7. RNA-seq libraries were prepared as described previously^25^. The quality and integrity of each library was checked with Agilent high-sensitivity DNA chips (Agilent Technologies, Santa Clara, CA, USA). Concentrations of sequencing libraries were quantified using Qubit fluorometer and Qubit dsDNA HS assay kit (Life Technologies, CA, USA). Three biological replications of each genotype (USVL531-MDR, USVL677-PMS) per time-point were used for 150 bp paired-end RNA-seq sequencing (Duke Center for Genomic and Computational Biology, Durham, NC, USA) with Illumina HiSeq 4000 sequencing system (Illumina, Inc., San Diego, CA, USA).

### RNA-seq data analysis

Pre-processing of the next-generation sequencing (NGS) data was performed using the tools on Biopieces (https://www.biopieces.org). Pair-end reads were interleaved to maintain their order during the pre-processing, the bad quality reads trimmed from both ends (min Phred quality score < 35, good quality stretch length > 3) and then both forward and reverse adaptor sequences removed from the reads. The remaining reads were then filtered out by length, keeping the ones having 30 or more nucleotides (for each pair). Lastly, local mean scores were calculated and only reads higher than 25 were retained for the study. High quality reads were mapped to the watermelon reference genome *Citrullus lanatus* subsp. *Vulgaris* cv. 97103 v1^24^ using the mem algorithm from Burrows-Wheeler aligner (a.k.a bwa)^26^. Samtools^27^ was used to compress the (SAM) files into BAM files. The mapping rates for each library are summarized (Fig. 3). Differential expression analysis was performed using edgeR^28^ package in R^29^ to quantify gene/transcript expression and detect novel transcripts. In summary, for differential expression analysis biological coefficients of variation (BCV) were calculated between samples and then quasi-likelihood or dispersion (fold change) around the BCV trend were estimated using glmQLFTest function in edgeR. After that, differentially expressed genes selected as both having two fold or more changes and consistent among the replicates. Lastly, we used FDR ratio of < 0.05 to filter the most significant differentially expressed genes.

### Gene annotation, classification of DEGs into functional categories and KEGG analysis

The Gene Ontology (GO) classification of all the differentially expressed genes DEGs/ transcripts were categorized into broader GO classes using the GO enrichment and GO gene classification tools available in the Cucurbit Genomics Database https://cucurbitgenomics.org/pwyenrich and Blast2GO server (https://www.blast2go.com/)^30^. The KEGG pathway analysis was done using the KOBAS 3 database https://kobas.cbi.pku.edu.cn/kobas3.

### Whole-genome resequencing and comparative SNPs and InDel analysis

For whole-genome resequencing, high-quality genomic DNA was extracted from leaves of four week old watermelon seedlings (USVL531-MDR, USVL677-PMS, and four RILs: R-201, R-202, R-203 and R-204, having 1–2 fully expanded true leaves using the Qiagen DNeasy Plant Mini Kit as suggested by the manufacturer’s instructions (Qiagen, Hilden, Germany). DNA-seq libraries were prepared as per Novogene technology (https://en.novogene.com/next-generation-sequencing-services/animal-and-plant-genome/animal-plant-genome-sequencing) using HiSeq X Ten platform, paired-end 150 bp reads. The quality and integrity of each library was checked with Agilent high-sensitivity DNA chips (Agilent Technologies, Santa Clara, CA, USA). Concentrations of sequencing libraries were quantified using Qubit fluorometer and Qubit dsDNA HS assay kit (Life Technologies).

To identify sequence variants including SNPs and insertions/deletions (INDELs), Fastq files generated by Novogene for each RIL line were mapped to the watermelon (*Citrullus lanatus* subsp. *Vulgaris* cv. 97103)^24^ reference genome (www.icugi.org/pub/genome/watermelon/97103/v1/), using mem algorithm in Burrows-Wheeler aligner^26^. Resulting SAM files converted into BAM using samtools^27^. Haplotype Caller from GATK’s v3.7^31^ used for variant calling with default parameters. Variants were filtered based on SNPs and INDELs with minimum allele count (-c1), variant allele count for given position (e.g. biallelic SNPs -M2) and saved into zipped vcf files for each line using bcftools view^32^. A union table of all the vcf files created using bcftools isec for easier downstream analysis. Sliding window analysis was performed using a custom script written in Perl on all the SNPs and INDELs separately with slide size of 20 k and window size of 1 M nucleotides. Final data was visualized using Circos for each line^33^.

### Cleaved amplified polymorphic sequence (CAPS) analysis

In order to validate the single nucleotide polymorphism (SNP) associated with *ClaPMR2* locus (*Citrullus lanatus* PM Resistance gene, *Cla019831*), PCR based CAPS (cleaved amplified polymorphic sequence) marker was designed to genotype the parent lines: USVL531-MDR and USVL677-PMS, F2’s population of USVL531-MDR × USVL677-PMS cross and the PM resistant introgressed lines R-201, R-202, R-203 and R-204. The primers were designed with a program called dCAPS Finder 2.0 (https://helix.wustl.edu/dcaps/dcaps.html). Primers used for *ClaPMR2*-CAPS were 5′-GAAGACCAGAGAATTCGTGTGACTACC-3′ and 5′-CTGTCCATAAAGTAAATGTTCGGGTGAG-3′. The genotype at the *ClaPMR2* locus was determined by amplifying a 509 bp region flanking the SNP nucleotide base position # 1,474 (C-T; Arg-STOP), and digesting the amplified product with TaqI restriction enzyme. Sequence analysis of *ClaPMR2* locus revealed a substitution of C to T in USVL677-PMS genome which abolishes the restriction enzyme site for Taq 1 in the *ClaPMR2* amplified DNA of USVL677-PMS line, whereas in USVL531-MDR, PM resistant line, digested PCR fragment products of 477 bp and 32 bp were obtained. For restriction digestion assay, twenty microlitres of the PCR product were digested in 1 × CutSmart Buffer with 1 U of TaqI restriction enzyme (New England Biolabs, Beverly, MA) incubated for 2 h in a 65 °C water bath, followed by electrophoretic separation (2.5% agarose, 1 × TAE buffer pH 8.0, 1 h, 120 V).

## Results

### USVL531-MDR (R) and USVL677-PMS (S) responded distinctly to powdery mildew inoculation

**Figure 2:**
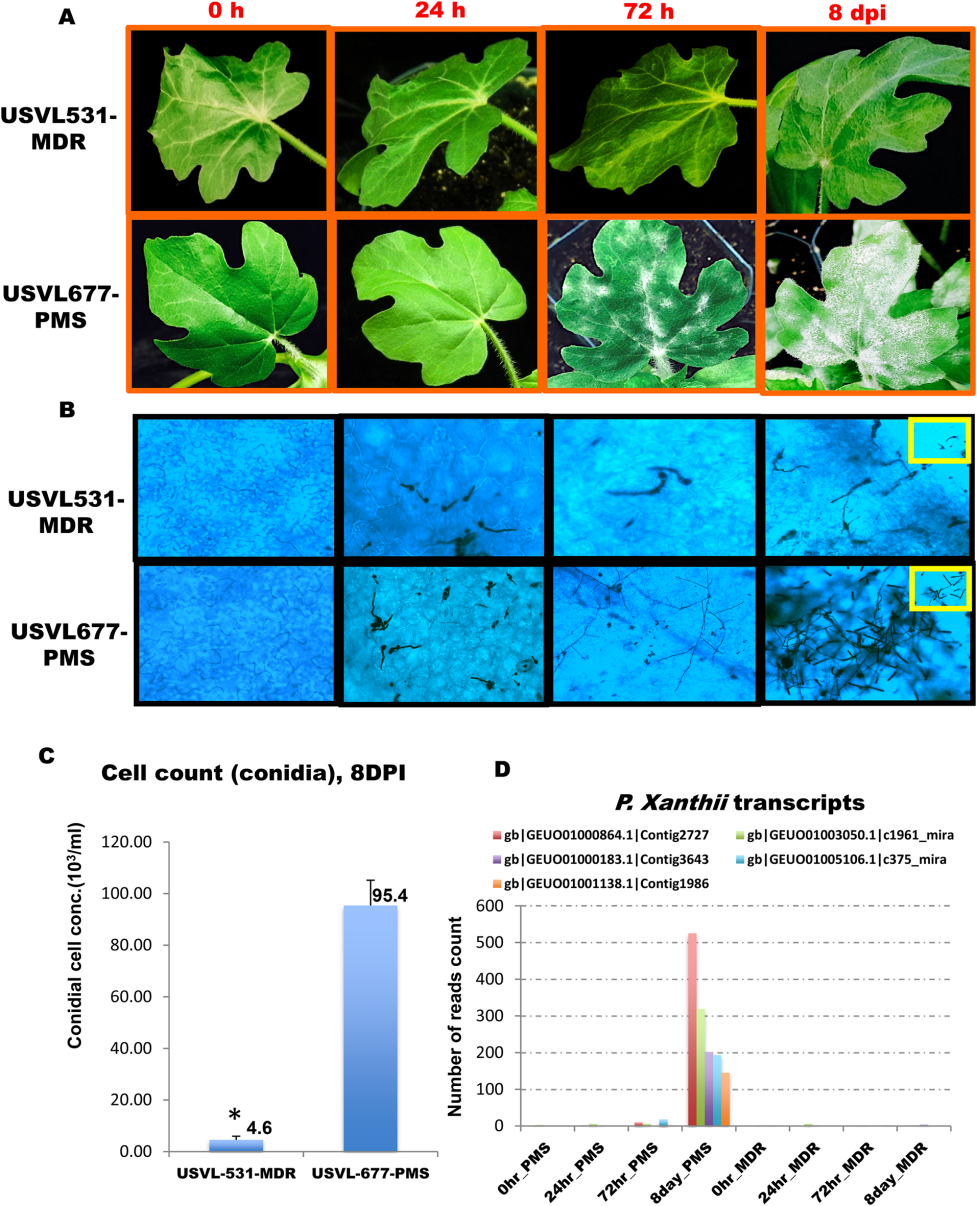
Time course PM infection process in USVL531-MDR and USVL677-PMS watermelon lines*.* (**A**) PM conidial development on leaves of 3–4 week old watermelon plant USVL531-MDR: Powdery mildew resistant line; USVL677-PMS: powdery mildew susceptible line at 0 h, 24 h, 72 h and 8 dpi with powdery mildew fungus; (**B**) Trypan-blue stained hyphae collected from watermelon lines: USVL677-PMS & USVL531-MDR at 0 h, 24 h, 72 h and 8 dpi with powdery mildew fungus; (**C**) Bar graph representing PM conidial count on leaves of USVL677-PMS, USVL531-MDR at 8 dpi. Asterisks indicate data statistically significant from that of susceptible line (USVL677-PMS; *P* < 0.05). (**D**) Bar graph representing *P. xanthii* secretome transcript^50^ counts on leaves of USVL677-PMS, USVL531-MDR at 0 h, 24 h, 72 h and 8 dpi. Asterisks indicate data statistically significant from that of susceptible line (USVL677-PMS; *P* < 0.05).

To compare the PM-induced characteristic disease symptoms between USVL531-MDR (PM resistant/ multiple disease resistant) and USVL677-PMS (PM susceptible) watermelon lines, we conducted microscopy as well as observation of visual symptom development (white powdery growth) starting from germination of conidia to hyphal development and proliferation during the eight-day time period (8 DPI) of the study. Visual symptom development on adaxial leaf surfaces are presented in Fig. 2A at 0 h, 24 h, 72 h and 8 dpi. A significant difference between USVL531-MDR and USVL677-PMS in response to PM over time (0 h to 8 DPI) was observed. USVL531-MDR exhibits high level of resistance to PM caused by *P. xanthii* as compared to USVL677-PMS. At 72 h post inoculation visual conidial chains were observed on USVL677-PMS while no disease symptoms were observed on the resistant line, USVL-531-MDR. At 8 DPI, the hypocotyls, cotyledons and true leaves of USVL531-MDR continued to exhibit resistance to PM when compared to USVL677-PMS on which severe PM growth and abundant development of conidia were observed (Fig. 2A–C). On microscopic observation of trypan-blue stained PM pathogen inside the epidermal cells of USVL531-MDR and USVL677-PMS over time (0 h to 8 DPI), the leaves of USVL531-MDR showed a significant reduction in conidial germination and hyphal growth and development as compared to USVL677-PMS (Fig. 2B,C). The conidia were able to germinate, form appressoria and secondary hyphae at 24 h on USVL677-PMS while no appressoria or conidia germination were observed in the resistant line. In addition, we observed significant increased levels of *P. xanthii* transcripts/ secretomes in USVL677-PMS (PM susceptible line) at 8 dpi (50 ×) as compared to USVL531-MDR (PM resistant line) (Fig. 2D). Taken together, USVL531-MDR (R) and USVL677-PMS (S) responded distinctly to powdery mildew inoculation.

### Transcriptomic reprogramming of plant defense signaling network between resistant (USVL531-MDR) and susceptible watermelon (USVL677-PMS) in response to *P. xanthii* infection

**Figure 3:**
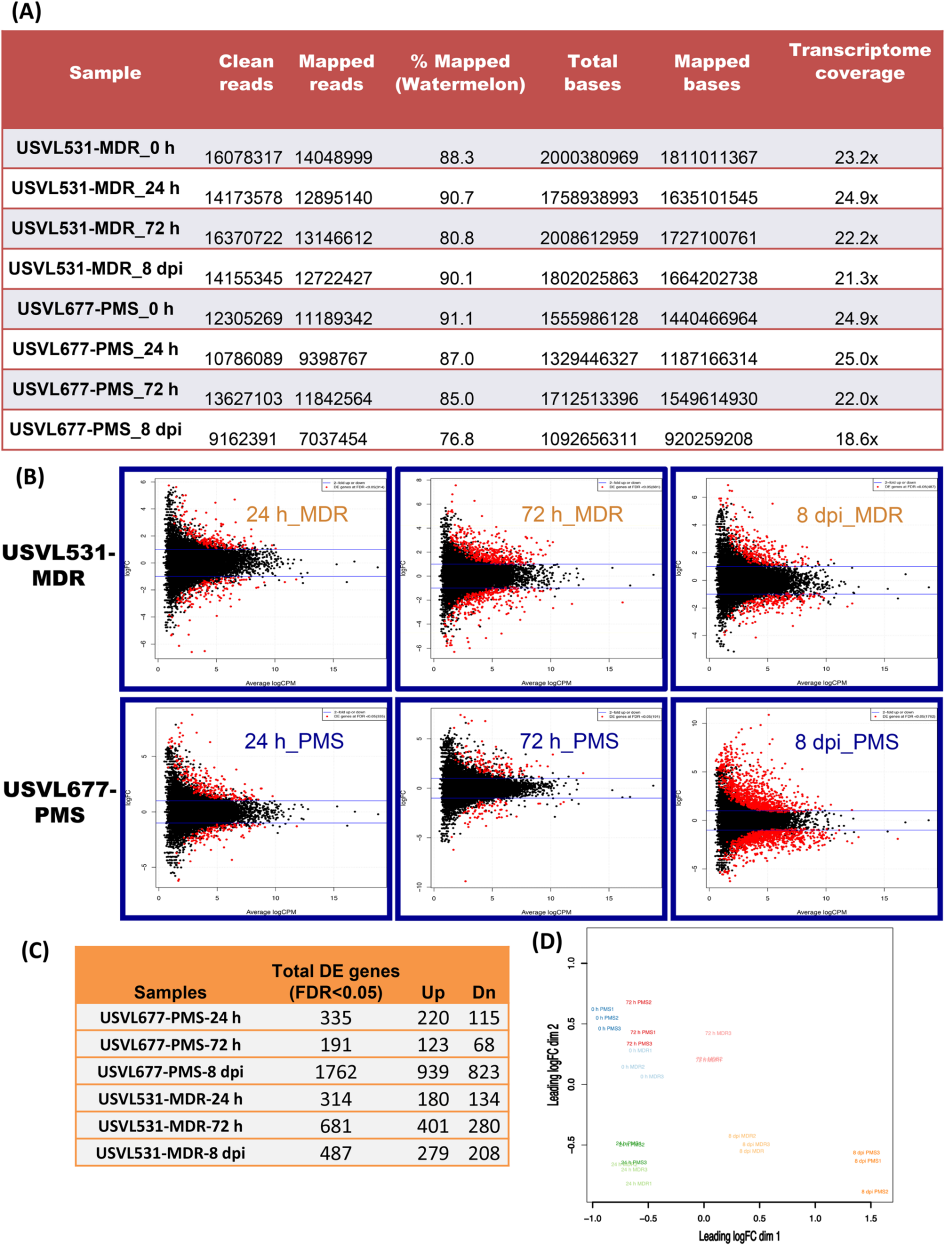
Processed RNA-seq data. (**A**) Total number of clean, mapped reads (watermelon) and bases of each mRNAseq sample (three replicates) following PM inoculation collected from watermelon lines: USVL677-PMS & USVL531-MDR at 0 h, 24 h, 72 h and 8 dpi. (**B**) Distribution of differentially expressed genes (DEGs) for each mRNAseq sample following PM inoculation collected from watermelon lines: USVL677-PMS & USVL531-MDR at 0 h, 24 h, 72 h and 8 dpi. The DEGs are shown in red logFC > 1, *P* value < 0.05 and FDR ratio of < 0.05 for each gene in each pair-wise comparison of each time points with uninoculated 0 h as control. The red dots highlight transcripts of positive and negative values of log2 Fold Change (logFC), indicating that the sequences were upregulated and downregulated at each time point. The black dots indicate non-differentially expressed genes. The genes names, fold changes, and *p* values, FDR values for up- and down-regulated DEGs were listed in Table S1. (**C**) Table showing total DEGs, upregulated and downregulated genes in response to PM in USVL677-PMS & USVL531-MDR at 24 h, 72 h and 8 dpi. (**D**) Multidimensional scaling (MDS) of all replicate samples.

**Figure 4:**
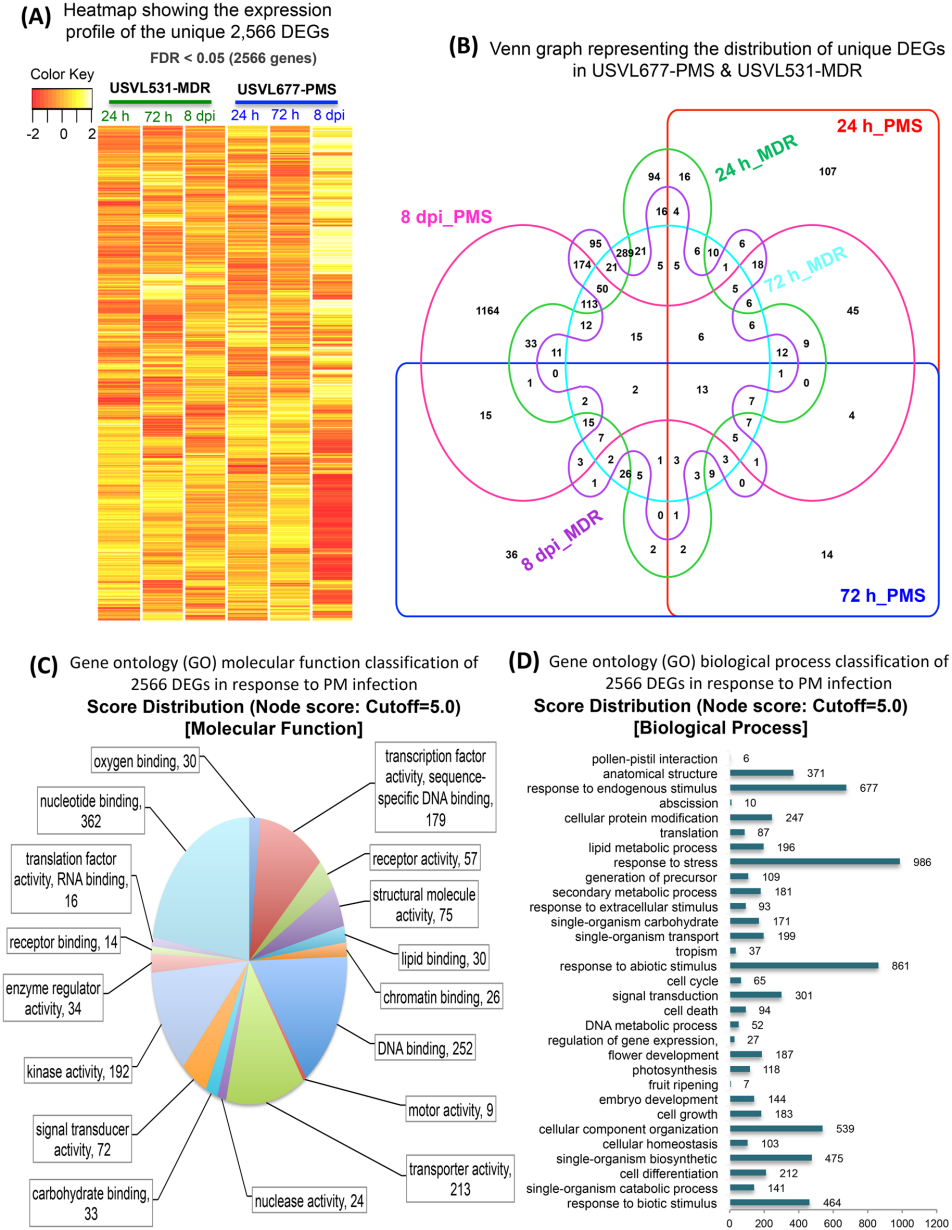
Expression profile and Gene ontology classification of unique 2,566 DEGs. (**A**) Heatmap showing the expression profile of the unique 2,566 DEGs in response to PM in USVL677-PMS & USVL531-MDR at 24 h, 72 h and 8 dpi. (**B**) Venn graph representing the distribution of unique DEGs in USVL677-PMS & USVL531-MDR at 24 h, 72 h and 8 dpi. VENNTURE^51^ software version 1.1.0.2 was used to generate the venn graph. (**C**) Gene ontology (GO) molecular function classification of 2,566 DEGs in response to PM infection. (**D**) Gene ontology (GO) biological process classification of 2,566 DEGs in response to PM infection. Blast2go software was used to generate the GO annotations (https://www.blast2go.com/)^30^.

Genome-wide gene expression profile was done by utilizing RNA-seq on PM inoculated (10^5^ conidia ml^−1^) watermelon leaves of a susceptible line USVL677-PMS, and this was compared to a resistant line, USVL531-MDR (PM-resistant) at 0 h, 24 h, 72 h and 8DPI. Approximately 9 to 16 million reads were generated from each RNA-seq library with a median of 14 million reads. Most of the reads mapped (77% to 91%) to the draft watermelon (*Citrullus lanatus* subsp. *Vulgaris* cv. 97103 v1^24^) genome sequence, with transcriptome coverage in the range of 19X to 25X (Fig. 3A). A total of 3,770 differentially expressed genes (DEGs) were identified during compatible and incompatible interactions with *P. xanthii,* out of which 2,566 DEGs were uniquely expressed. The compatible interaction resulted in distinct plant gene activation (> twofold unique transcripts, 335:191:1762 :: 1:3:8 DPI) as compared to incompatible interaction (> twofold unique transcripts, 314:681:487 :: 1:3:8 DPI) (Fig. 3B,C, Table S1). Host genotype effects (USVL531-MDR vs USVL677-PMS) were observed more clearly for *P. xanthii* infection at 72 h and 8DPI; 681 genes were differentially expressed in PM inoculated USVL531-MDR as compared to uninoculated control plants while only 191 genes were differentially expressed in the susceptible USVL677-PMS plants at 72 h. Contrasting PM response was observed during late stage infection process (8DPI) with severe PM growth and abundant development of secondary mycelia occurs in USVL677-PMS plants with 1,762 DEGs as compared to USVL531-MDR. Heatmap (Fig. 4A, red/yellow heatmap) and venn diagram (Fig. 4B) analysis of unique 2,566 DEGs between PM inoculated resistant USVL531-MDR and the susceptible USVL677-PMS revealed that each genotype have unique effect on transcript changes during PM infection process from 24 h to 8DPI with a maximum of unique 1,164 genes at 8DPI in USVL677-PMS having severe PM and conidial growth in USVL677-PMS plants (Fig. 2A–C).

Further, 2,566 unique DEGs between USVL531-MDR and USVL677-PMS were grouped into GO terms based on their predicted molecular functions and biological process (Fig. 4C,D), along with the number of expressed genes. Interestingly, 27 genes (Table S2) were observed to be regulator of gene expression, providing evidence of key determinants in the PM disease cycle.

Based on Heatmap and venn diagram (Fig. 4A,B) analysis of unique 2,566 DEGs, We observed significant differences in regulation of DEGs, 68.6% of all 2,566 DEGs (with 939 up-regulated and 823 down-regulated) during compatible USVL677-PMS- *P. xanthii* interaction at 8DPI, indicates the potential molecular mechanisms associated with pathogen virulence in the absence of innate plant defense response, resulting in severe disease symptoms in above-ground parts of the susceptible genotype than resistant genotype USVL531-MDR. In order to investigate the biological functions and molecular signaling events associated with compromised in resistance, the up-regulated (939) and down-regulated (823) DEGs identified during compatible USVL677-PMS- *P. xanthii* interaction at 8DPI, the DEGs were annotated to the GO database under three groups including biological process (BP), molecular function (MF) and cellular component (CC) (Supplementary Fig. S1).

Among BP, “response to stress”, “response to abiotic stimulus”, “response to endogenous stimulus”, “cellular component organization” and “response to biotic stimulus” were the dominant terms at 8DPI. Among MF, “binding”, “catalytic activity”, “protein binding”, “transcriptase activity”, “hydrolase activity”, “small molecule binding, sequence-specific DNA binding” and “kinase activity” were significantly enriched terms. Among CC, “cell”, “cell part”, “intra cellular organelle”, “cytoplasm”, “membrane”, “plastid” and “nucleus” were significantly enriched terms.

The DEGs from compatible interaction at 8DPI were further analyzed using KEGG functional enrichment (Supplementary Fig. S2 and Table S3). There were ninety-three (93, upregulated) and hundred one (101, downregulated) significantly enriched pathways including “metabolic pathways”, “biosynthesis of secondary metabolites”, “plant-pathogen interaction”, “plant hormone signal transduction”, “phenylpropanoid biosynthesis”, “MAPK signal pathway” “carbon metabolism” and “photosynthesis”. However, on comparative KEGG pathway enrichment analysis on *P. xanthii* inoculated USVL531-MDR and USVL677-PMS plants (Supplementary Fig. S3, Table S4). we observed metabolic pathway associated with TCA cycle was the most enriched pathway associated with plant defense against *P. xanthii*. This has been confirmed by our ongoing unpublished data to identify and study metabolic differences between the leaves of PM-susceptible (USVL677-PMS) and PM-resistant watermelon (USVL531-MDR) lines after 8 days post inoculation with *P. xanthii* conidia. In addition, the KEGG enriched pathway associated with “plant-pathogen interaction” (Supplementary Fig. S3) was mostly composed of AvrA10 effector mediated WRKY transcription factors (*Cla018026*), putative CC-NBS LRR disease resistance protein (*Cla007904*), as well as calmodulin-like Protein (*Cla010322*, *Cla020738*, *Cla021803*, *Cla021582*), heat shock protein HSP90 (*Cla022413*) calcium-binding protein and salicylic acid (SA) mediated upregulation enhanced disease susceptibility 1 (*EDS1*, *Cla019156*) and pathogenesis-related protein 1a (*PR1a*, *Cla001623*). We also observed fungal PAMP (Avr9) associated up-regulation of calcium dependent protein kinases (CDPKs), respiratory burst oxidase-like protein (*Cla017196*), mitogen-activated protein kinases and WRKY transcription factors regulating plant defense against *P. xanthii* in USVL531- MDR. However, we observed predominant role of RPM1 interacting protein 4 (RIN4, *Cla014936*), Receptor protein kinase-like protein (*Cla001497*), Receptor-like kinase (*Cla015191*), Ethylene-responsive transcription factor 4 (*Cla013424*), WRKY transcription factors (*Cla008104*, *Cla004431*, *Cla021207*, *Cla018026*), calmodulin and calcium dependent protein kinases as major player regulating the signaling events associated with *P. xanthii* -USVL677- PMS interaction. All these transcriptional changes provide a better understanding of the signaling pathway(s) that regulate the infection-related development of *P. xanthii* in watermelon.

**Figure 5:**
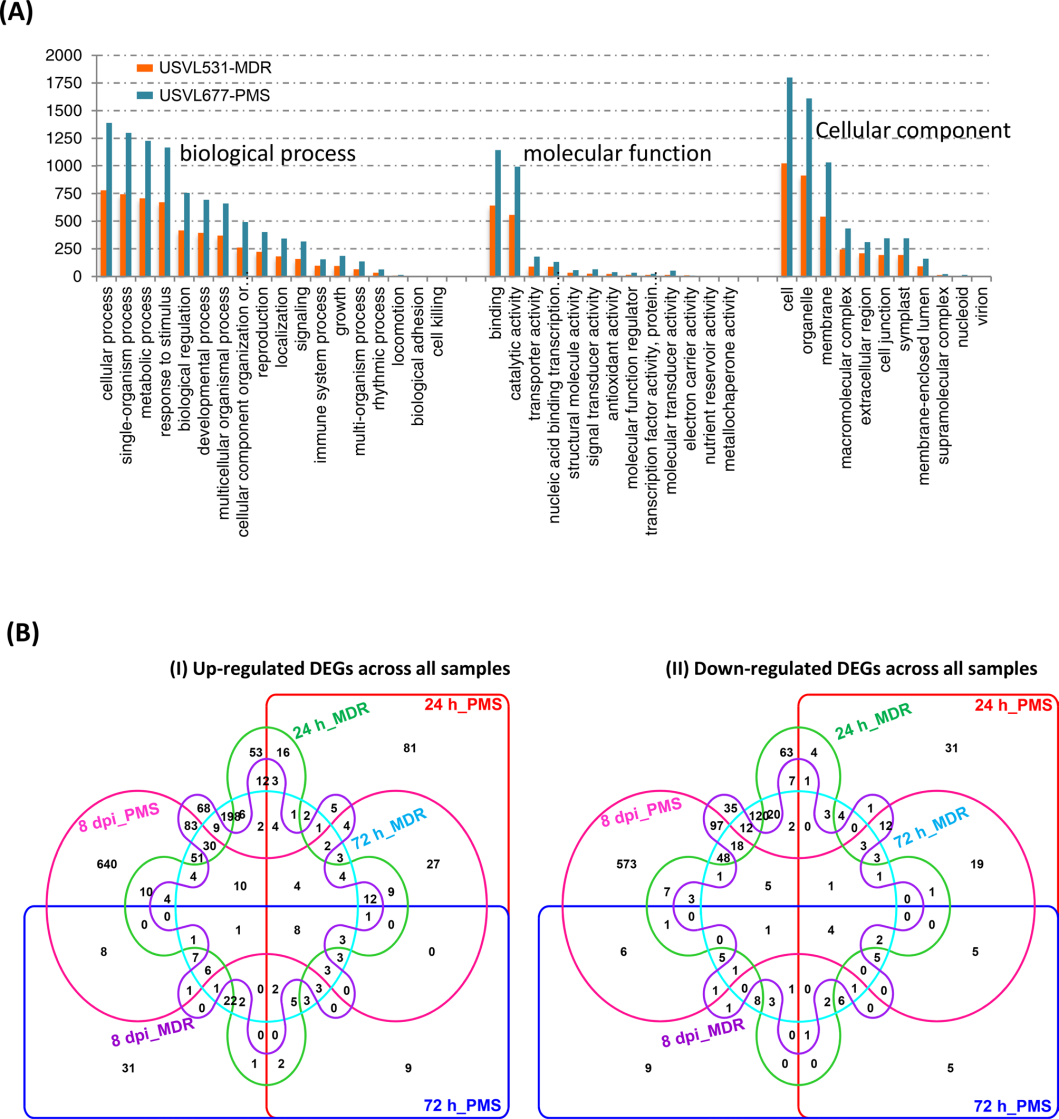
Comparative distribution of 2,566 unique DEGs between USVL531-MDR and USVL677-PMS. (**A**) Comparison of DEGs involved in biological process, molecular function and cellular compartmentalization in USVL677-PMS and USVL531-MDR. (**B**) Venn graph representing the distribution of upregulated & downregulated DEGs in USVL677-PMS & USVL531-MDR at 24 h, 72 h and 8 dpi. VENNTURE^51^ software version 1.1.0.2 was used to generate the Venn graph.

Comparative analysis of 2,566 unique DEGs between USVL531-MDR and USVL677-PMS revealed that most DEGs are involved in biological process related to cellular process (779, 1,389), single-organism process (743, 1,298), metabolic process (707, 1,226), and response to stimulus (671, 1,166) (Fig. 5A, Fig. S4). In terms of molecular function, most DEGs are classified under binding (641, 1,143), and catalytic activities (556, 992) (Fig. S5), with cellular (1,022, 1,799), organelle (912, 1,610), and membrane proteins (541, 1,031), are major cellular components.

Further comparison of all up-regulated 2,142 DEGs and down-regulated 1,628 DEGs across all samples during the course of infection process revealed a co-regulation of certain hormonal, MAP kinases, Ca^2+^ mediated signaling pathway genes and many stress responsive genes (Fig. 5B). We also observed temporal expression of up-regulated DEGs 53:2:68 :: 24 h:72 h: 8DPI specific to incompatible interaction while 81:31:640 :: 24 h:72 h: 8DPI DEGs up-regulated specific to compatible interaction. All this suggests that very few DEGs are activated during early stage of PM infection and appressoria formation. Further, our results show an overlap of activated basal defense response genes during compatible and incompatible interaction. All these suggest that plants respond to pathogen invasion by activating an array of similar defense mechanisms. In addition, there is a host specific interaction of PM pathogen during early stage of conidia germination to appressoria development between USVL531-MDR and USVL677-PMS and the affected DEGs during early stage of infection determines resistance or susceptibility between the two cultivars.

**Figure 6:**
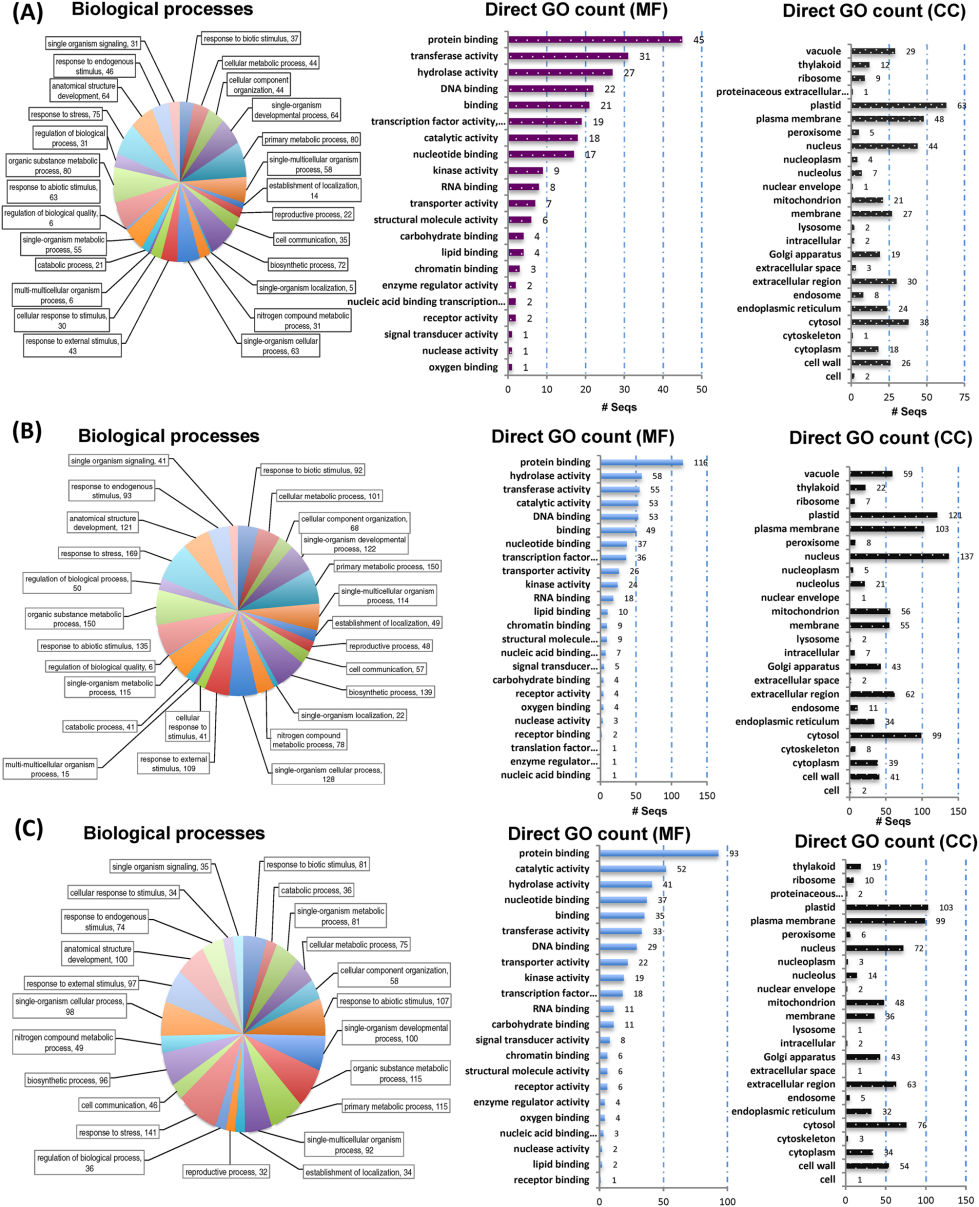
Distribution of up-regulated differentially expressed transcripts/genes (DEGs) involved in biological processes, molecular functions and cellular components in USVL531-MDR at (**A**) 24 h (**B**) 72 h and (**C**) 8 dpi. Blast2go software was used to generate the GO annotations (https://www.blast2go.com/)^30^.

**Figure 7:**
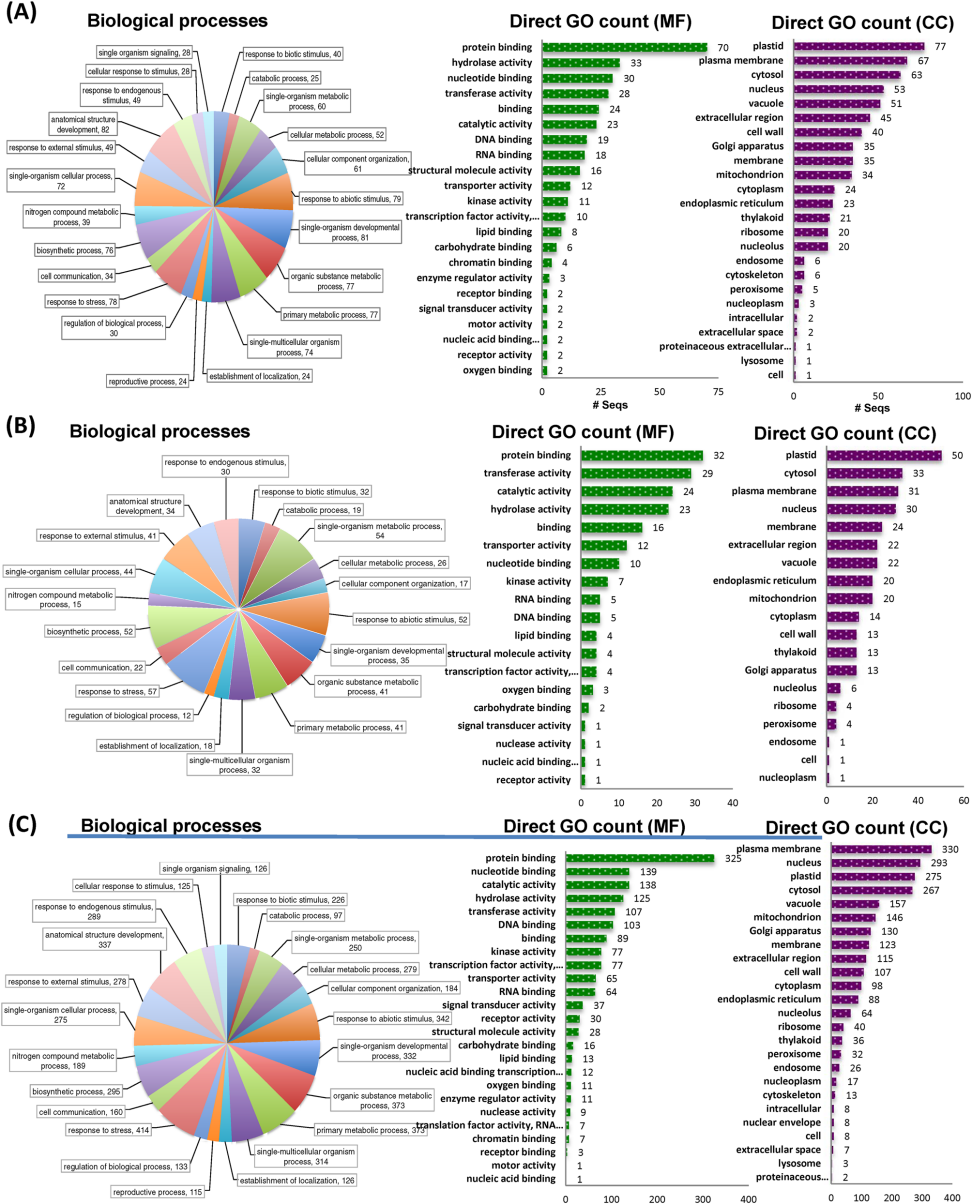
Distribution of up-regulated differentially expressed transcripts/genes (DEGs) involved in biological processes, molecular functions and cellular components in USVL677-PMS at (**A**) 24 h (**B**) 72 h and (**C**) 8 dpi. Blast2go software was used to generate the GO annotations (https://www.blast2go.com/)^30^.

**Figure 8:**
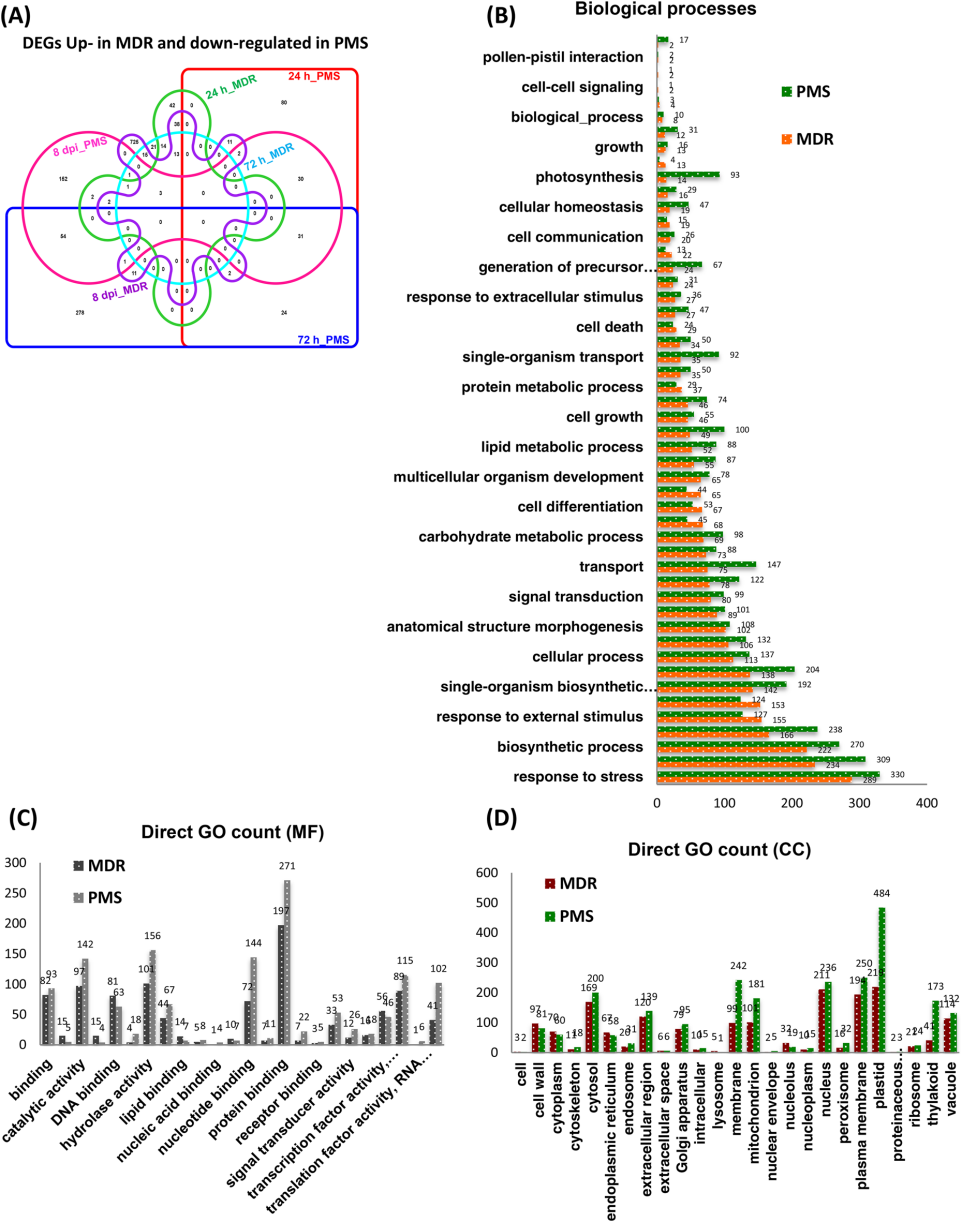
Comparative analysis of the distribution of up-regulated DEGs involved in biological processes, molecular functions and cellular components in USVL531-MDR as compared to down-regulated DEGs in USVL677-PMS after PM inoculation. (**A**) Venn graph representing the distribution of upregulated DEGs in USVL531-MDR as compared to downregulated DEGs in USVL677-PMS at 24 h, 72 h and 8 dpi. VENNTURE^51^ software version 1.1.0.2 was used to generate the venn graph. (**B**) Gene ontology (GO) biological process classification of DEGs in response to PM infection. (**C**) Gene ontology (GO) molecular function classification of DEGs in response to PM infection. (**D**) Gene ontology (GO) cellular components classification of DEGs in response to PM infection.

Previous studies have focused on well-known upstream signaling pathways, but our transcriptomic study provides in detail of the downstream regulators during compatible and incomapatible interaction (Figs. 6 and 7). Host specific upregulated DEGs during incompatible and compatible interaction were grouped into GO terms based on their predicted biological process along with the number of expressed genes involved in particular biological process, molecular function and based on cellular components at particular time of infection, 24 h (Figs. 6A and 7A), 72 h (Figs. 6B and 7B), and 8 dpi (Figs. 6C and 7C). Based on the expression profile analysis during incompatible interaction (*P. xanthii*-USVL531-MDR), the majority of induced defense pathway genes are involved primarily in metabolic processes (80, 150, 115), with molecular function protein binding (45, 116, 93) with plastid as cellular component (63, 121, 103). In response to *P. xanthii* interaction, the expression of several resistance proteins, both TIR-NBS-LRR and CC-NBS-LRR were also significantly changed during compatible and incompatible interaction (Table S5).

In order to investigate the constitutive defense mechanism associated with USVL531-MDR, we did comparative analysis of the distribution of up-regulated DEGs involved in biological processes, molecular functions and cellular components in USVL531-MDR as compared to down-regulated DEGs in USVL677-PMS after PM inoculation (Fig. 8A). We observed 80:278:152 genes up-regulated in a time kinetic manner at 24 h, 72 h, 8DPI post *P. xanthii* inoculation specific to USVL531-MDR while 42:21:725 genes down-regulated in a time kinetic manner at 24 h, 72 h, 8DPI post *P. xanthii* inoculation specific to USVL531-MDR. However, during incompatible interaction in USVL531-MDR, we observed a common induced defense signaling pathway associated with 31 genes (Table S6) during the *P. xanthii* infection at 24 h, 72 h, 8DPI. While 13 common genes were constitutively down-regulated in compatible USVL677-PMS- *P. xanthii* interaction at 24 h, 72 h, 8DPI. Three genes (*Cla018352*, *Cla016180*, *Cla007029*) were found to be commonly up-regulated at 8DPI in USVL531-MDR while down-regulated at all time points in USVL677-PMS. In our annotation results of distribution of DEGs, of the 50 most over-represented GO terms, stress response related ones (GO:0006950) appeared highest in both USVL531-MDR (289) and USVL677-PMS (330) lines (Fig. 8B; Table S7.1, S7.2) which supports the hypothesis that both compatible and incompatible plant-pathogen interactions showed some common signaling pathways. In our expression data, for the 860 up-regulated DEGs in incompatible reaction, the most over-represented GO terms in molecular function were “protein binding” (GO:0005515), and “hydrolase activity”(GO:0016788) accounting for 23% (197 genes), 12% (101 genes) respectively of the annotated terms (Fig. 8C). In addition to protein binding and hydrolases, we also observed differential expression of other defense regulation genes, such as genes related to DNA binding (81), transcription factor (56), signal transducer activity (12), receptor activity (7) and receptor binding (3) (Fig. 8C). We observed that the majority of the regualtory DEGs were found to encode plastidial, plasma membrane, nuclear, membranous and cytosolic proteins (Fig. 8D). However, nearly 1.5% of sequences were categorized by GO annotation as comprising extracellular region/space (126 genes in USVL531-MDR), supporting the hypothesis of extracellular recognition of *P. xanthii* with host encoded proteins to initiate resistance signaling against the pathogen.

**Figure 9:**
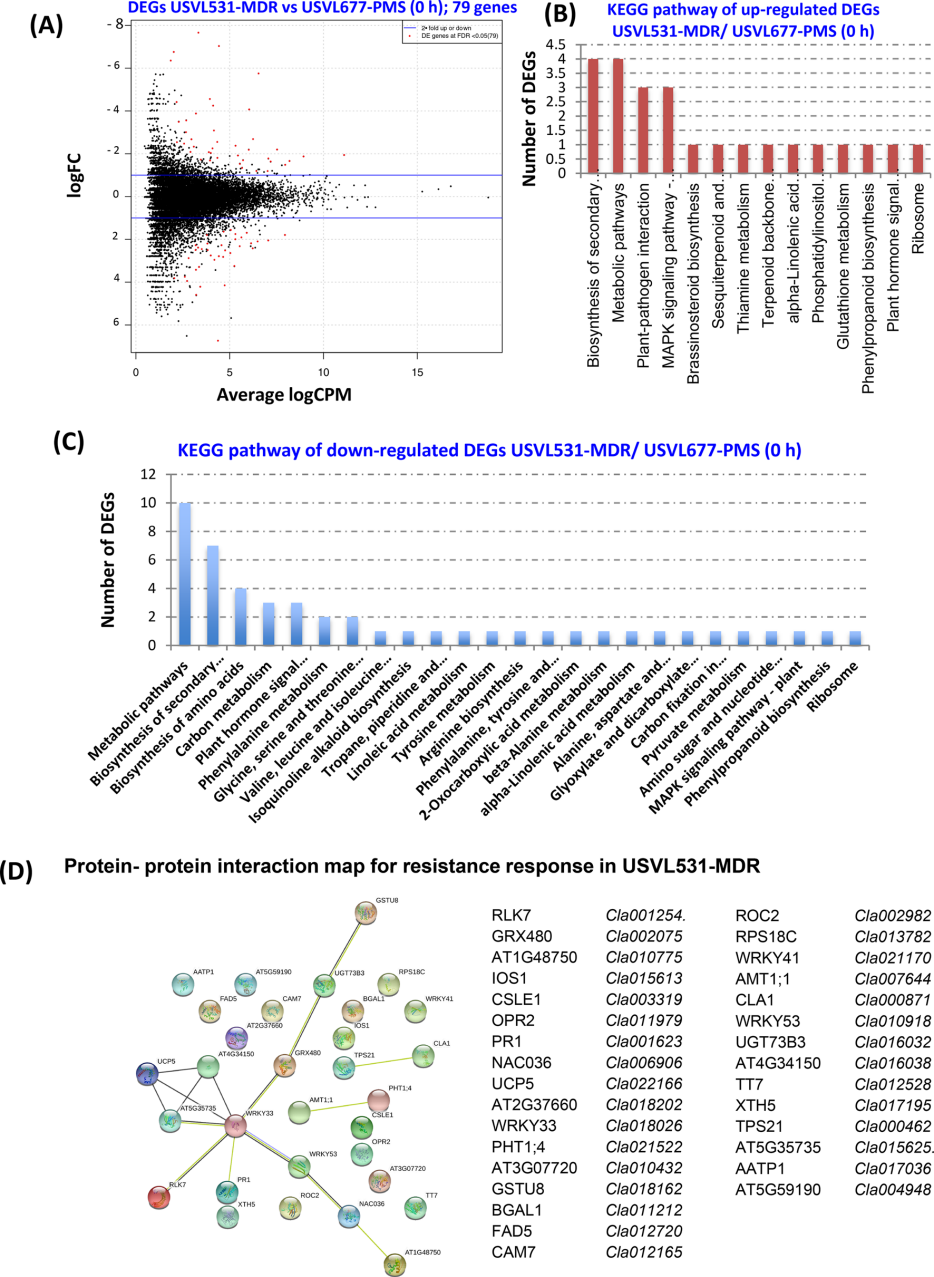
Expression profile and KEGG enrichment analysis of DEGs in Multiple Disease Resistance Line (MDR) as compared to PM susceptible line (PMS) at 0 h. (**A**) Scatter plot of DEGs USVL531-MDR (0 h) vs USVL677-PMS (0 h); 79 genes. The red dots highlight transcripts of positive and negative values of log2 Fold Change (logFC), (**B**) KEGG pathway of up-regulated DEGs USVL531-MDR/ USVL677-PMS (0 h) (**C**) KEGG pathway of down-regulated DEGs USVL531-MDR/ USVL677-PMS (0 h) (**D**) Protein- protein interaction map for resistance response in USVL531-MDR.

Since USVL531-MDR (R) and USVL677-PMS (S) responded distinctly to powdery mildew inoculation, we also tested if any of the regulated DEGs may have been derived from two accessions. We identified a total of 79 differentially expressed genes (37 DEGs up-regulated and 42 DEGs down-regulated) at 0 h un-inoculated samples when PM resistant USVL531-MDR was compared to PM susceptible USVL677-PMS (Fig. 9A and Table S9). Comparative KEGG gene enrichment analysis (Fig. 9B,C) showed that majority of DEGs perturbed were associated with metabolic pathways, biosynthesis of secondary metabolites and f plant-pathogen interaction factors contributing to basal defense response in USVL531-MDR as compared to compromised USVL677-PMS which were further confirmed by the protein–protein interactome map of 37 up-regulated DEGs (Fig. 9D) using STRING database https://string-db.org/ with *Arabidopsis thaliana* as the reference organism. Taken together, we observed a constitutive defense pathway do exists in USVL531-MDR affecting the salicylic acid pathway and along with the up-regulated plant cellulose synthase complex (Fig. S6, Table S10) which could be providing basal defense in USVL531-MDR.

### Molecular mapping of PM resistance loci in watermelon

Four red fleshed PM resistant RIL introgressed lines (R-201, R-202, R-203 and R-204), developed from a cross between USVL531-MDR × USVL677-PMS, showed no visible PM symptoms for 5 generations and were resequenced along with the parental lines. Sequence variations including single nucleotide polymorphisms (SNPs) and insertions and deletions (InDels) were identified by aligning all reads to the watermelon (*Citrullus lanatus* subsp. *Vulgaris* cv. 97103) reference genome (www.icugi.org/pub/genome/watermelon/97103/v1/). Table 1 provides a detailed summary on the whole genome resequencing data on parent lines (USVL531-MDR and USVL677-PMS) and the introgressed lines (R-201, R-202, R-203 and R-204). A total of 108.06 million, 123.00 million, 47.58 million, 29.96 million, 46.60 million and 57.39 million paired-end reads were obtained for USVL531-MDR, USVL677-PMS, R-201, R-202, R-203 and R-204 respectively. The average sequence depth was 42-fold in USVL531-MDR, 49-fold in USVL677-PMS, 25-fold in R-201, 16-fold in R-202, 24-fold in R-203 and 30-fold in R-204 introgressed line. A total of 492,103, 264,170, 331,733, 335,950, 286,536 and 299,279 SNPs, as well as 129,546, 74,113, 79,916, 81,830, 72,828 and 75,021 InDels were identified in USVL531-MDR, USVL677-PMS, R-201, R-202, R-203 and R-204, respectively, by comparing each individually with the *Citrullus lanatus* subsp. *Vulgaris* cv. 97103 reference genome assembly. A total of 421,727, 406,136, 499,575, 459,749, 453,138 SNPs, as well as 99,507, 115,292, 127,136, 120,748 and 120,171 InDels, were identified in USVL677-PMS, R-201, R-202, R-203 and R-204, respectively, by comparing each individually with USVL531-MDR. However, we observed reduction in both SNPs and InDels in R-201 (260,641, 69,641), R-202 (254,718, 70,205), R-203 (212,694, 63,107) and RIL204 (225,181, 65,474) lines by comparing each individually with USVL677-PMS (Tables 2 and 3).

**Table 1:** Summary of whole-genome re-sequencing (WGR) data of multiple disease resistant watermelon line: USVL531-PMR; PM susceptible watermelon line: USVL677-PMS; introgressed RILs: R-201, R-202, R-203, R-204.

Mapping statistics	PMR (USVL531)	PMS (USVL677)	R-201	R-202	R-203	R-204
Total reads	123,711,017	138,059,639	55,830,914	34,607,712	58,224,410	69,226,371
Mapped reads	123,231,114	137,610,938	54,485,300	34,337,658	55,136,172	67,721,273
Properly paired	108,061,484	123,001,650	47,581,686	29,968,198	46,609,024	57,397,362
Average depth	42.4957	48.5718	24.5452	15.6176	23.9883	29.9511
Q30 percentage	81.95%	85.14%	82.98%	85.01%	79.16%	80.99%

**Table 2:** Result summary of comparative SNP analysis among USVL531-PMR, USVL677-PMS, R-201, R-202, R-203, R-204 and the watermelon reference genome 97103.

	PMR (USVL531)	PMS (USVL677)	R-201	R-202	R-203	R-204	97103
PMR (USVL531)	0	421,727	406,136	499,575	459,749	453,138	492,103
PMS (USVL677)		0	260,641	254,718	212,694	225,181	264,170
R-201			0	252,809	221,573	211,154	331,733
R-202				0	49,414	114,753	335,950
R-203					0	83,915	286,536
R-204						0	299,279
97103							0

**Table 3:** Result summary of comparative insertions and deletions (INDELS) analysis among USVL531-PMR, USVL677-PMS, R-201, R-202, R-203, R-204 and the watermelon reference genome 97103.

	PMR (USVL531)	PMS (USVL677)	R-201	R-202	R-203	R-204	97103
PMR (USVL531)	0	99,507	115,292	127,136	120,748	120,171	129,546
PMS (USVL677)		0	69,641	70,205	63,107	65,474	74,113
R-201			0	48,866	45,704	43,195	79,916
R-202				0	9,002	18,775	81,830
R-203					0	16,267	72,828
R-204						0	75,021
97103							0

**Figure 10:**
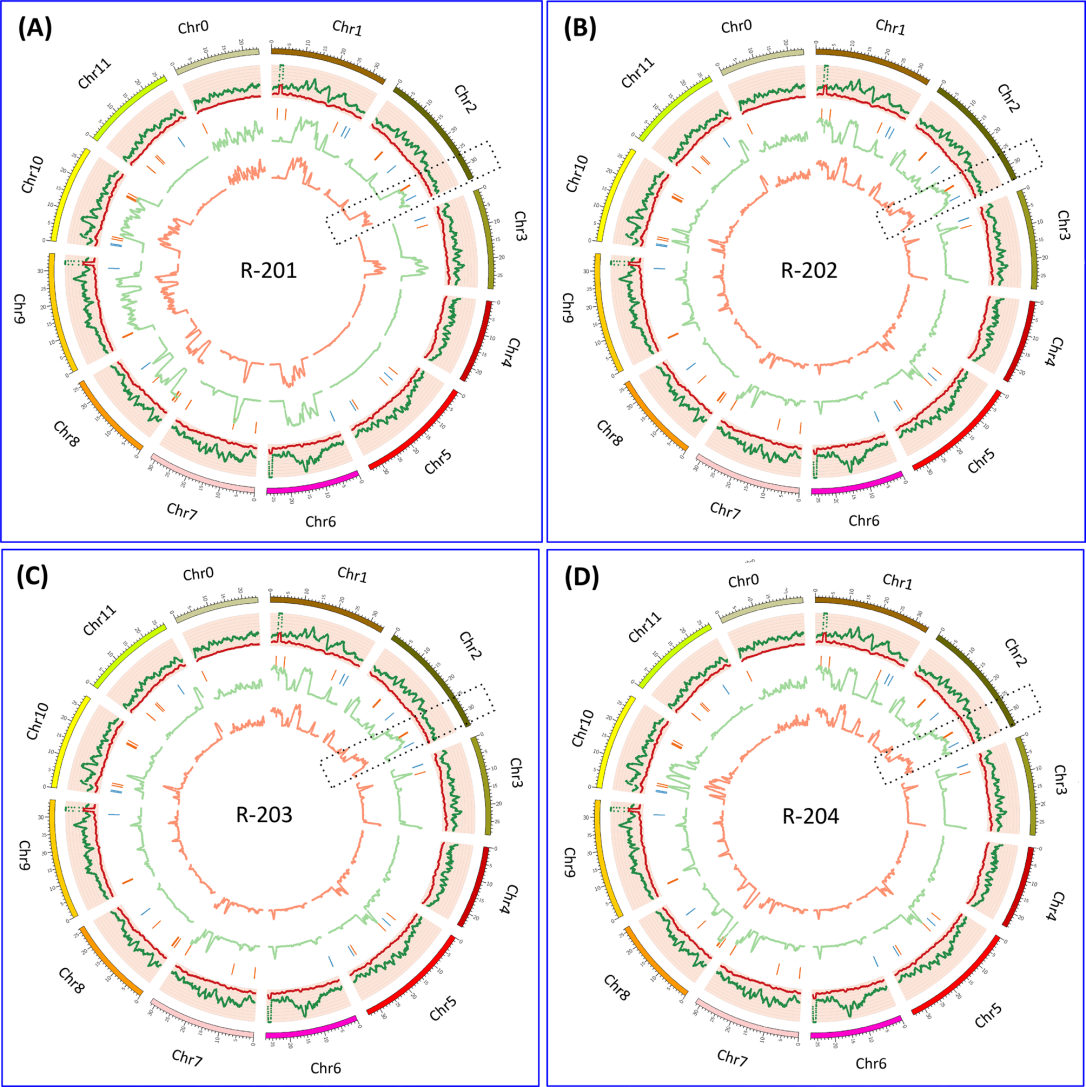
Circos plots showing the distribution of SNPs, InDels, NBS-LRR and Mlo genes in PM resistance introgressed lines. The outer ring represents the eleven watermelon chromosomes. The scatter plot inside this ring shows the distribution of homozygous SNPs (dark green) and InDels (red) between (**A**): *Citrullus lanatus* subsp. *Vulgaris* cv. 97103 and R-201; (**B**): *Citrullus lanatus* subsp. *Vulgaris* cv. 97103 and R-202; (**C**): *Citrullus lanatus* subsp. *Vulgaris* cv. 97103 and R-203; (**D**): *Citrullus lanatus* subsp. *Vulgaris* cv. 97103 and R-204. The green line represents the genotype percentage of SNPs and orange line indicates the genotype percentage of InDels in CSK lines from the donor parent USVL531-MDR along the chromosomes. The NBS-LRR and Mlo gene locations are represented in orange and blue lines respectively along the chromosomes. Black dotted rectangle area represents the chrs 2 region with increased genotype percentage of SNPs and InDels with 7 NBS-LRR and one Mlo gene. Circos-0.69-6 software was used to generate the circos plot (https://circos.ca/software/download/circos/)^33^.

**Figure 11:**
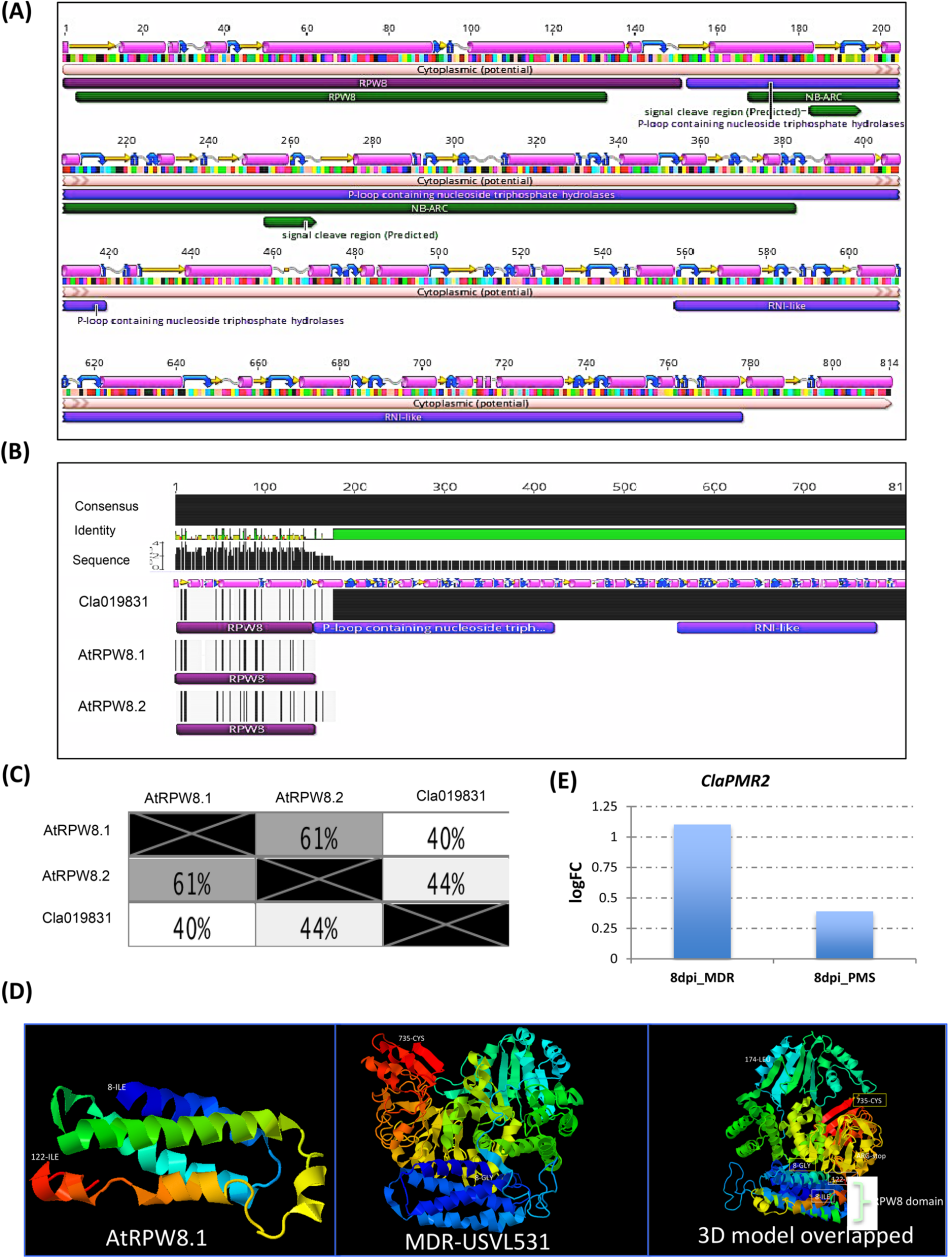
Predicted Secondary Structure of watermelon *ClaPMR2*. (**A**) Open reading frame of *ClaPMR2* protein with predicted secondary structure and transmembrane domains using EMBOSS 6.5.7 (Geneious Prime v2019.2.1, https://www.geneious.com/prime/). The ORF consists of the conserved for the N terminal RPW8-EHM targeted motif. The Predicted secondary structure of alpha helix, beta strand, coil and turn are presented in purple cylinders, yellow arrows, grey sinusoids and blue curved arrow. Detailed information of individual sequences is presented in Table S8. (**B**) Comparative secondary structure and transmembrane domains analysis of *Arabidopsis* RPW8.1, RPW8.2 with the watermelon *ClaPMR2* using EMBOSS 6.5.7 (Geneious Prime v2019.2.1, https://www.geneious.com/prime/). (**C**) Comparative percentage of sequence (aa) similarity of *ClaPMR2*/ Cla019831 with AtRPW8.1 and AtRPW8.2 domain. (D) 3D modeling with structural overlap of the RPW8 domain of *Arabidopsis thaliana* (AtRPW8.1) with the N-terminal domain of watermelon, Cla-RPW8 (USVL531-MDR). Geneious Prime v2019.2.1, https://www.geneious.com/prime/) was used to generate the 3D structure model. ClaPMR2 expression level at 8dpi in USVL531-MDR and USVL677-PMS.

**Figure 12:**
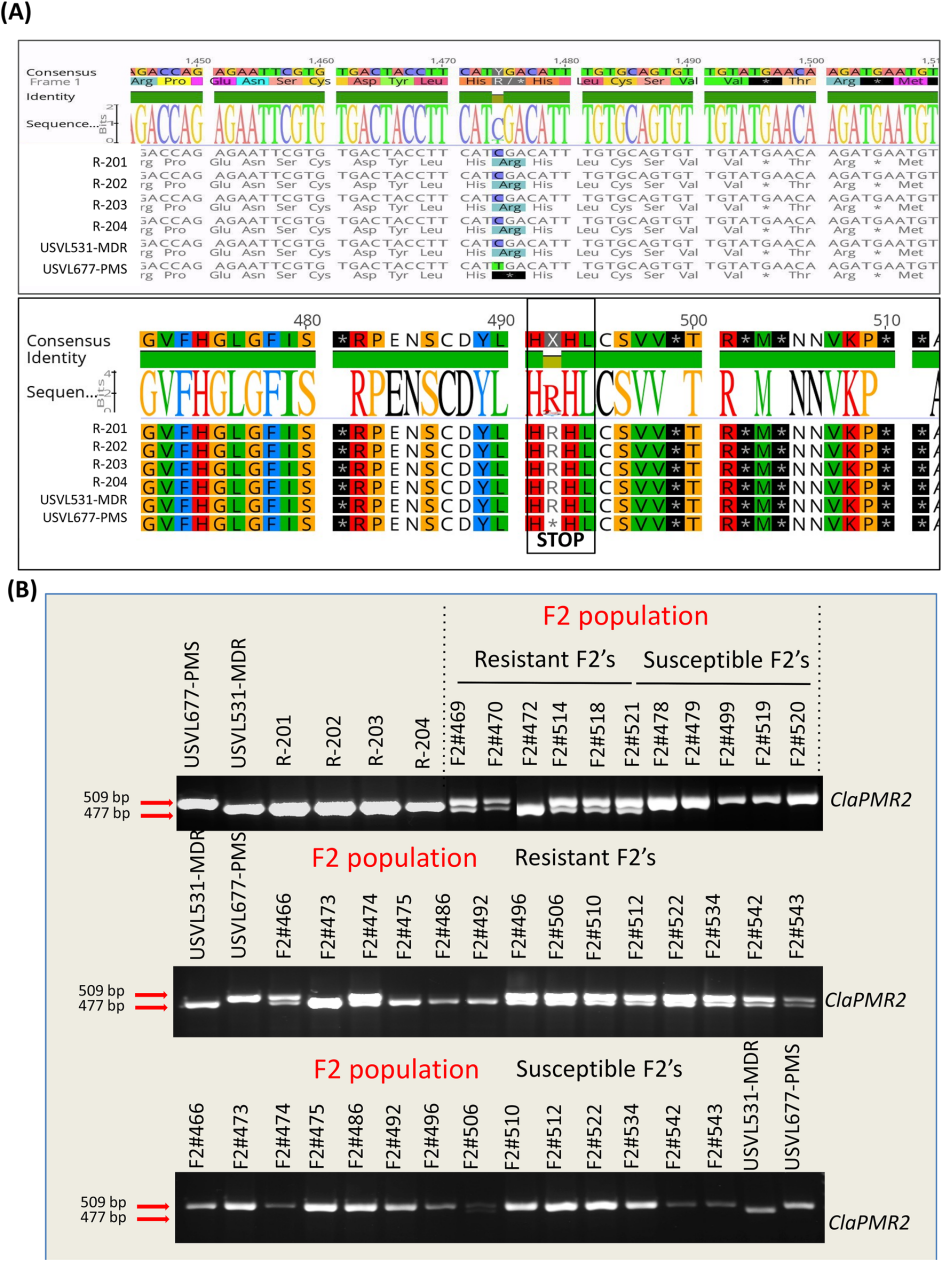
SNPs derived CAPS validation on Parent, F_2_ and RIL introgressed watermelon lines using DNA Agarose gel. (**A**) Comparative nucleotide and amino acid consensus sequences with predicted secondary structure alignment of *ClaPMR2* encoding protein in Parent (USVL531-MDR & USVL677-PMS), and RIL introgressed watermelon lines (R-201, R-202, R-203, R-204). Black line rectangle area HRH (C-T; Arg-STOP), represents the location of SNP region with substitution position Arg-STOP codon in (*ClaPMR2, Citrullus lanatus PM Resistance gene*). (**B**) DNA gel electrophoresis showing the CAPS marker analysis on *ClaPMR2 gene* in parent lines: USVL531-MDR & USVL677-PMS; RILs: R-201, R-202, R-203 and R-204 and F_2_ populations. Since the resistant phenotype is dominant, we observed *ClaPMR2 loci*, cosegregated with the resistant locus in heterozygous and homozygous individuals with PM resistance lines.

**Figure 13:**
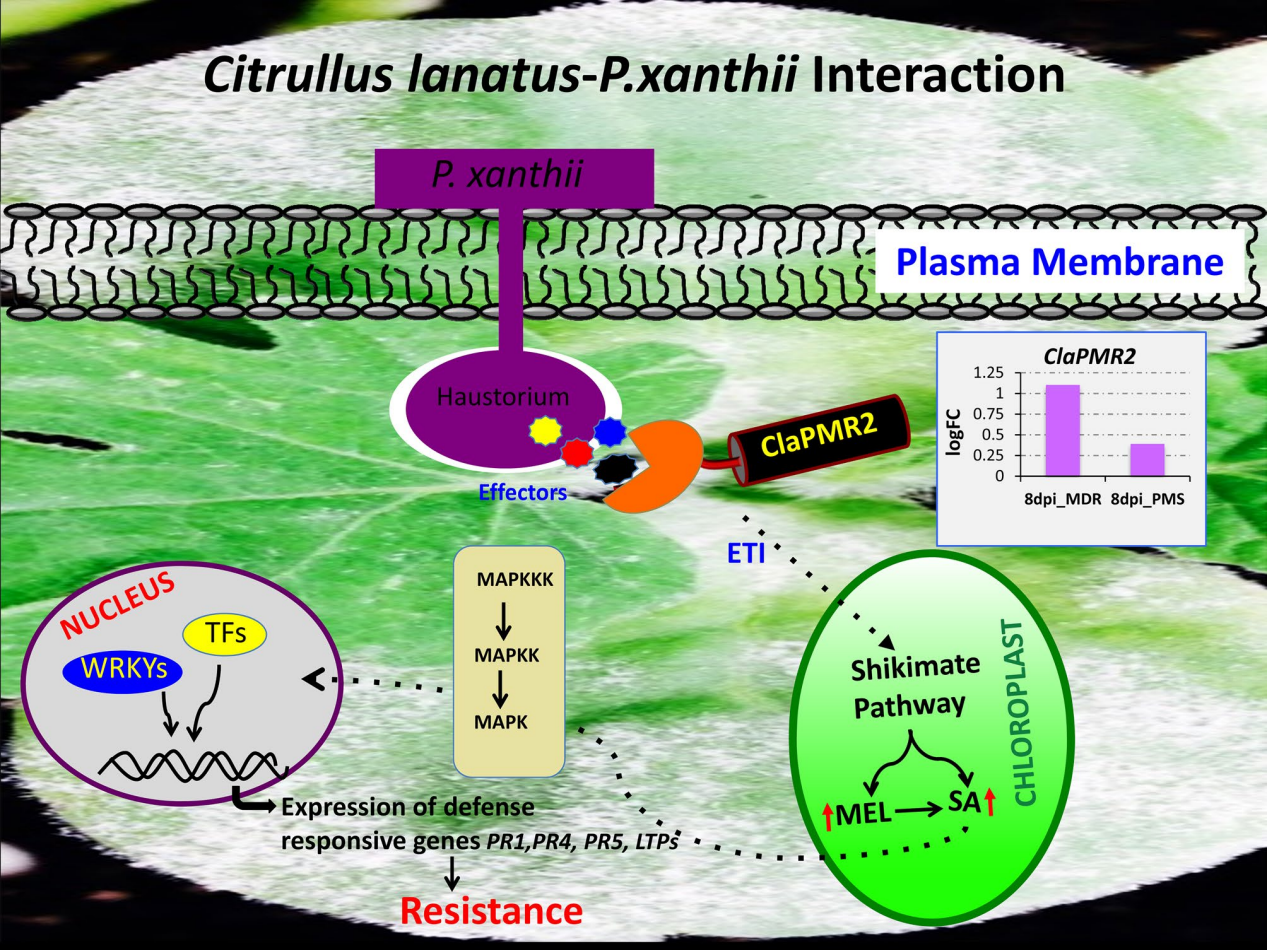
Proposed model of *ClaPMR2* mediated resistance signaling in watermelon in response to *P. xanthii*. During the infection process, the plant pathogenic fungi, *P. xanthii* develop highly specialized structures known as haustoria to secrete specific effector molecules. These effectors are recognized by the sophisticated plant specific intracellular immune receptors (NBS-LRRs, *ClaPMR2*) to activate the ETI mediated plant defense response by activating shikimate pathway to produce elevated levels of melatonin (MEL)^21^ and salicylic acid (SA) in chloroplast. The increased SA levels further potentiate downstream signaling by triggering nuclear gene expressions of various pathogenesis-related (PR1, PR5) and other defense related genes providing resistance to pathogen. During host–pathogen interaction the pathogenic fungi can alters expression of diverse defense related genes from initial germination of conidia to hyphal development and proliferation during the 8-day time period activating diverse intra- and extra-cellular resistance proteins downstream to *ClaPMR2* MEL (M): melatonin; ETI: effector triggered immunity; TFs: transcription factors; MAPKKK: map kinase kinase kinase; MAPKK: map kinase kinase; MAPKKK: map kinase; NBS-LRR: nucleotide-binding site leucine-rich repeat.

Recent studies with GBS and GWAS analysis by Wu et al.^34^, on 1,147 *Citrullus* PIs has indicated the probable existence of diverse SNPs for PM resistance against *P. xanthii* (race 1 W and 2 W), 12 SNPs in chromosome 2 and 21 SNPs in multiple chromosomes chrs 1 (3), chrs 3 (3), chrs 4 (2), chrs 7 (3), chrs 8 (4), chrs 9 (4), chrs 10 (2). However, due to the presence of diverse races of the cucurbit PM pathogen *P. xanthii* across the US^35,36^ and lack of resistant cultivars to specific races, identification and mapping of resistance loci to all races has been difficult. The comparative analyses of homozygous SNPs and InDels introgressed into the four RIL lines from the multi-disease resistant line USVL531-MDR enabled us to identify a single genomic region with potential genes/ alleles associated with PM resistance on chromosome 2 (25.2–27.1 MB, 175 annotated genes). Generally, plant resistance to fungal pathogens requires recognition of the haustorium secreted avr (AVR) proteins by the plant resistance proteins (R) to activate disease resistance response. We identified 57 candidate NBS-LRR resistance proteins (both TIR: Toll-interleukin receptor and CC: coiled-coiled receptors) based on the annotated genes/protein sequence encompassing the entire watermelon draft genome sequence (www.icugi.org/pub/genome/watermelon/97103/v1/). In addition, by utilizing conserved amino acid sequence similarity from *Arabidopsis thaliana* and *Brassica oleraceae* we identified 14 genes with homology to Mildew resistance locus o (Mlo), in watermelon. Mlo’s are known to confer broad-spectrum post penetration haustorium-targeted resistance against diverse PM fungus^37,38^. We mapped the location of all the NBS and Mlo-like proteins in watermelon genome *Citrullus lanatus* subsp. *Vulgaris* cv. 97103 (Fig. 10, circo plot) and identified 10 NBS-LRR and 2 Mlo genes in chromosome 2 genome (Table 4). However, we identified seven NBS-LRR genes {*Cla019863*, Chr2 : 26383499 .. 26388744 (−); *Cla019857*, Chr2 : 26432098 .. 26437657 (+), *Cla019856*, Chr2 : 26439873 .. 26444126 (+); *Cla019855*, Chr2 : 26449200 .. 26453033 (+); *Cla019854*, Chr2 : 26456943 .. 26459976 (−); *Cla019844*, Chr2 : 26582380 .. 26589679 (−); *Cla019831*, Chr2 : 26750001 .. 26753327 (−)} and one Mlo gene {Cla008753, Chr2 : 31285860 .. 31292414 (+)} in the region of increased genotype percentage of homozygous SNPs and InDels in all RIL lines donated from USVL531-MDR line. This comparative genome sequence analysis of each of the seven NBS-LRR genes from watermelon lines (USVL531-MDR, USVL677-PMS, R-201, R-202, R-203 and R-204) led to the identification of homozygous SNPs donated from USVL531-MDR into the RIL lines. One of the NBS-LRR protein (R) *Cla019831* {Chr2 : 26750001 .. 26753327 (−), with > twofold increase in transcriptome in USVL531-MDR at 8 dpi (Fig. 11E) } showed sequence similarity to the *Arabidopsis thaliana* resistance protein, RPW8 ( Resistance to PM 8)^39^ which is known to provide resistance to a broad range of PM pathogens. We compared the sequence (nucleotide and amino acid) similarity between *Cla019831* (Cla-RPW8) with AtRPW8.1 and AtRPW8.2 domain (Fig. 11A,B; Table S8) and was well conserved with 40–44% (Fig. 11C) sequence identity within the RPW8 domain which was further supported by 3D modeling with structural overlap of the RPW8 domain of *Arabidopsis thaliana* (AtRPW8.1) with the the N-terminal domain of watermelon, Cla-RPW8 (USVL531-MDR) (Fig. 11D). We observed four SNPs at nucleotide position # 84 (G-A; Arg-Arg), # 1474 (C-T; Arg-STOP), # 1818 (A-G; Arg-Val), # 2607 (T-A; Ser-Arg) when compared with resistant lines USVL531-MDR, R-201, R-202, R-203 and R-204 and the PM susceptible line USVL677-PMS. Based on SNPs identification, PCR based CAPS (cleaved amplified polymorphic sequence) marker were designed for the # 1474 substitution position Arg-STOP codon (*ClaPMR2, Citrullus lanatus PM Resistance gene*) with appropriate enzyme site to genotype the parent line: USVL531-MDR and USVL677-PMS along with the PM resistant introgressed lines: R-201, R-202, R-203 and R-204 (Fig. 12A). The CAPS marker *ClaPMR2*, co-segregated with the resistant locus in USVL531-MDR, R-201, R-202, R-203 and R-204 as compared to susceptible line USVL677-PMS (Fig. 12B). Based on the phenotypic screening of F_2_ populations (P1: USVL531-MDR × P2: USVL677-PMS), the resistant locus was found to be a dominant gene (data unpublished). Since bulked segregant analysis (BSA) was considered an effective method for identifying markers linked to a target gene. We selected 20 individual plants with distinct resistant and 20 individual plants with susceptible phenotypes from an F2 population, based on PM disease index scoring for CAPS marker analysis. The resistant plants were rated < 1 (2% or less leaf area covered with PM) on a 0–10 scale and the susceptible were rated > 8 (> 91% of leaf area covered by PM with abundant visible conidia). Since the resistant phenotype is dominant, we observed *ClaPMR2 loci*, co-segregated perfectly with the resistant locus in heterozygous and homozygous individuals with PM resistance in all 20 lines tested (Fig. 12C) which was further confirmed by CAPS marker analysis on randomly selected seventy additional USVL531-MDR × USVL677-PMS segregating F2’s with genotypic ratio of R: 15 (USVL531-MDR homozygous):: heterozygous, H/− : 37:: S:18 (USVL677-PMS homozygous). A previous report indicated the requirement of *AtRPW*8 to broad-range of PM pathogens in *Arabidopsis thaliana*, however, resistance role of *Cla-PMR2* in watermelon-PM interaction has not been studied. In a previous study we evaluated disease response of eleven (11) PM isolates collected from various cucurbit plants and different states in the US on cotyledons of both USVL531-MDR and USVL677-PMS^35,36^. All isolates grew and produced conidia on USVL677-PMS while USVL531-MDR was resistant to all isolates, supporting our hypothesis of the requirement of functional *ClaPMR2* in USVL531-MDR for resistance against broad-range of PM isolates similar to AtRPW8*.* To date no resistance gene has been mapped to PM resistance in watermelon. Notably, this is the first time an F_2_ breeding population, combined with transcriptomic and genome resequencing technology has been used for the mapping and screening of candidate genes for the watermelon PM resistance gene. This study will be valuable for *ClaPMR2* cloning and resistance breeding utilizing *ClaPMR2* gene as the PM resistance marker in watermelon. Further, we have proposed a model of ClaPMR2 mediated resistance signaling in watermelon in response to *P. xanthii* interaction (Fig. 13).

**Table 4:** List of NBS and MLO genes associated with chromosome 2.

Sl no.	Gene ID	Description	Chromosome position	logFC_24h	*P* value	logFC_72h	*P* value	logFC_8dpi	*P* value
1	Cla002071	MLO-like protein 9	Chr2 : 17236517 .. 17243715 (+)						
2	Cla019863	TIR-NBS disease resistance-like protein	Chr2 : 26383499 .. 26388744 (−)						
3	Cla019857	TIR-NBS disease resistance-like protein	Chr2 : 26432098 .. 26437657 (+)	0.30	0.65	0.70	0.28	1.38	0.02
4	Cla019856	TIR-NBS disease resistance-like protein	Chr2 : 26439873 .. 26444126 (+)						
5	Cla019855	TIR-NBS-LRR disease resistance protein	Chr2 : 26449200 .. 26453033 (+)						
6	Cla019854	TIR-NBS disease resistance-like protein	Chr2 : 26456943 .. 26459976 (−)						
7	Cla019844	Cc-nbs-lrr resistance protein	Chr2 : 26582380 .. 26589679 (−)	1.25	0.03	0.54	0.36	1.02	0.08
8	Cla019831 *ClaPMR2*	Disease resistance protein	Chr2 : 26750001 .. 26753327 (−)					1.10	0.01
9	Cla008753	MLO-like protein 3	Chr2 : 31285860 .. 31292414 ( +)	1.94	0.04	1.89	0.04	2.78	0.00
10	Cla006803	Cc-nbs-lrr resistance protein	Chr2 : 9631689 .. 9634916 (+)						
11	Cla006813	Cc-nbs-lrr resistance protein	Chr2 : 9860817 .. 9862469 (+)						
12	Cla006820	Cc-nbs resistance protein	Chr2 : 9989292 .. 9992234 (−)

## Discussion

Powdery mildew (PM) of cucurbits caused by *Podosphaera xanthii* is one of the common and most destructive pathogen on many vegetable crops including watermelon and other crops in the *cucurbitaceae* family. It can limit plant photosynthesis due to whitish conidial growth on cotyledons, true leaves, leaf petioles and stems, which leads to premature loss of foliage and, subsequently, affect fruit quality and yield. We have also observed PM development on premature and matured fruits affecting marketable quality and quantitative value^12^. Fungicides and resistant varieties are the major means of PM control. However, fungicides are costly, environmently unfriendly and often lead to evolution of insensitive/resistant races against specific fungicides. Recent studies by our lab^21^ have shown application of environmental-friendly immune inducer, melatonin can boost plant immunity and suppress pathogen growth (*P. xanthii* and *P. capsici)* in watermelon and other cucurbit crops, where fungicide resistance and lack of genetic resistance are of major concern. In order to enhance resistance breeding in commercial cultivated watermelon lines, identification of major gene regulators during PM susceptibility and resistance are important. In the present study, we provided insight on the differential regulation of various genes during compatible and incompatible interaction by transcriptomic analysis in a time course PM infection process in USVL677-PMS (PM susceptible, compatible) and USVL531-MDR (PM-resistant/ multiple disease resistant, incompatible) watermelon lines. The USVL531-MDR showed enhanced resistance to PM pathogen at all time points, whereas USVL677-PMS was compromised in immunity with severe PM growth and abundant development of conidia on hypocotyls, cotyledons and true leaves were observed at 8DPI. This corresponds with more DEGs at 8DPI in USVL677-PMS. Our time-course transcriptomic data uncovered diverse gene activation associated with novel signaling pathway(s) and cellular metabolism(s) during compatible and incompatible watermelon-PM (*P. xanthii*) interactions. Some unigenes were identified to be primarily involved in early resistance signaling events (24 h) while others function as late responsive genes (8 DPI). During early phase of PM infection and establishment, 16 common genes were differentially expressed in both USVL531-MDR and USVL677-PMS at 24 h. Two of the upregulated transcription factors (TFs); *Cla003748* (Trihelix TF)^40^ and *Cla021059* (BHLH TF)^41^ has been known previously to be involved in disease resistance mechanism against fungal pathogens. Another gene *Cla022082*, a WD-40-repeat family is repressed and may function, as platforms for assembly of protein complexes in initiation of early signaling events leading to either disease resistance or susceptibility. Based on the expression profile analysis, PM infection process also affects major sets of genes of molecular function classified under receptor binding/-activity, transcription factor activity, lipid binding, DNA/-nucleotide binding proteins and mitogen-activated protein kinase (MAPK) signaling cascade. Further, significant differences were observed in regulation of DEGs during compatible USVL677-PMS- *P. xanthii* interaction at 8DPI indicating the potential molecular mechanisms involved in pathogen virulence resulting in severe disease symptoms appeared in above-ground parts of the susceptible genotype than resistant genotype USVL531-MDR. Our previous reports have shown that plastidial metabolite, melatonin can provide immunity to plants in response to PM infection^21^. Furthermore, a set of receptor activity genes *Cla011362*, *Cla007393* (24 h); *Cla017206*, *Cla007393*, *Cla014681* (72 h); *Cla007280*, *Cla011362*, *Cla009990*, *Cla004534*, *Cla007393*, *Cla014681* (8 dpi), and receptor binding genes *Cla005698*, *Cla021470* (72 h), were also induced in response to PM infection. Since PM infection requires nuclear gene regulation to initiate the defense mechanism, we observed a large subset of genes involved in biotic stimulus (37, 92, 81) and cell communication (35, 57, 46) with molecular functions involved in DNA binding, transcription factor activity, signal transducer activity providing evidence of genes associated with downstream signaling cascade during PM-watermelon interaction. Some of these genes were identified as members of the pathogenesis-related transcriptional factor, BHLH transcription factor, MYC2 transcription factor, serine/threonine protein kinase gene family, which are significantly induced in USVL531-MDR and involved in direct nuclear regulation of genes involved in resistance signaling against *P. xanthii*. In response to *P. xanthii* interaction, expression of several resistance proteins, both TIR-NBS-LRR and CC-NBS-LRR were significantly changed. Taken together, our data suggests probable requirement of both types of resistance proteins/receptors in defense signaling against PM pathogen. In addition, our study also support the role of cultivar/ accession specific DEGs (a total of 79 DEGs; 37 up-regulated and 42 down-regulated) could be provididing innated defense in PM resistant USVL531-MDR watermelon line as compared to PM susceptible USVL677-PMS. Further, comparison of all up-regulated 2,142 DEGs and down-regulated 1,628 DEGs across all samples during the course of infection process revealed a co-regulation of certain hormonal, MAP kinases, Ca^2+^ mediated signaling pathway genes and many stress responsive genes. We observed eight up-regulated DEGs (*Cla018507, Cla016053, Cla011807, Cla006692, Cla017373, Cla021980, Cla010665, Cla017037*) to be constitutively expressed at all time points in both USVL531-MDR and USVL677-PMS.

In our expression data, for the 860 up-regulated DEGs in incompatible reaction, the most over-represented GO terms in molecular function were “protein binding” (GO:0005515), and “hydrolase activity” (GO:0016788). Previous studies have shown that often there is requirement for multiple, interdependent protein associations in pathogen recognition to initiate host mediated resistance signaling during plant-pathogen interaction. We identified proteins such as *Cla019888*, a mitogen-activated protein kinase kinase kinase 3; *Cla016199*, basic helix-loop-helix dna-binding family protein; *Cla019156*, enhanced disease susceptibility 1; *Cla000815*, cysteine-rich rlk (receptor-like protein kinase); Cla003005, snf1-related protein kinase; *Cla023088*, leucine-rich receptor-like protein kinase family proteins were constitutively active as signal transducer during *P. xanthii*- USVL531-MDR interaction and are known to be involved in biotic stress providing resistance to plants against the pathogen^42,43^. Hydrolases too have recently received increased attention in the context of plant defense response (https://www.luke.fi/en/blog/plants-strike-back-episode-3-hydrolases/). The hydrolases include most of the pathogenesis related (PR) family proteins and are important in context of plant defense response against diverse pathogenic fungi. PR-proteins are glycoside hydrolases with β-glucanase (PR2, PR5) and chitinase activities (PR3, PR4, PR8, PR11)^44–48^. We speculate that in watermelon-*P. xanthii* interactions, the host can either recognize cell components associated with the pathogen (β-1,3-glucan, chitin) activating the pathogen associated molecular patterns (PAMP)^49^ mediated defense signaling or induce genes related to protein binding in the host cytoplasm, further activating downstream regulatory genes (transcription factors, DNA binding proteins, kinase activity), a process similar to PMAP triggered immunity (PTI) and effector triggered immunity (ETI). Overall these findings will provide new insights to identify important gene/alleles of interest require for PM resistance/susceptibility and will serve as genetic resources to develop microsatellites/ SSR markers for molecular breeding program to develop PM resistant watermelon.

Further, to speed up/improve the efficiency of novel gene discovery associated with PM resistance to non-model specialty crops like watermelon we performed SNPs and InDEL analysis, utilizing four introgressed RIL lines and identified a predictive *ClaPMR2* gene locus involved in PM resistance, which reveals the association of conserved resistance (R) protein mediated PM resistance during *Citrullus lanatus*–*P. xanthii* interaction, that may involve multiple signal regulators and transducers in addition to carbohydrate metabolism, cell wall modifications and the hormone-signaling pathway. The key findings of this study will be useful to speed up the breeding program for PM resistance against *P. xanthii* in specialty crops such as watermelon and other cucurbits where fungicide resistance and lack of resistant genetic material are of major concern.

## Supplementary information

Supplementary Legends.

Supplementary Fig. S1.

Supplementary Fig. S2.

Supplementary Fig. S3.

Supplementary Fig. S4.

Supplementary Fig. S5.

Supplementary Fig. S6.

Supplementary Table S1.

Supplementary Table S2.

Supplementary Table S3.

Supplementary Table S4.

Supplementary Table S5.

Supplementary Table S6.

Supplementary Table S7.1.

Supplementary Table S7.2.

Supplementary Table S8.

Supplementary Table S9.

Supplementary Table S10.

## Data Availability

The datasets generated during and analysed during the current study are available in BioProject, at https://www.ncbi.nlm.nih.gov/sra/?term=prjna886666.
