# Author Correction: Elucidation of resistance signaling and identification of powdery mildew resistant mapping loci (*ClaPMR2*) during watermelon-*Podosphaera xanthii* interaction using RNA-Seq and whole-genome resequencing approach

**DOI:** 10.1038/s41598-022-24544-4

**Published:** 2022-11-28

**Authors:** Mihir Kumar Mandal, Haktan Suren, Chandrasekar Kousik

**Affiliations:** 1 U.S. Vegetable Laboratory, USDA, ARS, 2700 Savannah Highway, Charleston, SC 29414 USA; 2 Department of Biology, Claflin University, Orangeburg, SC 29115 USA; 3 ORISE Participant Sponsored by the U.S. Vegetable Laboratory, USDA, ARS, 2700 Savannah Highway, Charleston, SC 29414 USA; 4 Department of Forest Resources and Environmental Conservation, Virginia Tech, Blacksburg, VA 24061 USA

Correction to: *Scientific Reports* https://doi.org/10.1038/s41598-020-70932-z, published online 20 August 2020

The Data Availability section in the original version of this Article was omitted. The Data Availability section now reads:

Data availability:

The datasets generated during and analysed during the current study are available in BioProject, at https://www.ncbi.nlm.nih.gov/sra/?term=prjna886666.

The original Article has been corrected.

