# Supplementary material for: Elucidation of resistance signaling and identification of powdery mildew resistant mapping loci (ClaPMR2) during watermelon-Podosphaera xanthii interaction using RNA-Seq and whole-genome resequencing approach
Source: Sci Rep. 2020 Aug 20;10:14038. doi: 10.1038/s41598-020-70932-z (PMC7441409; doi:10.1038/s41598-020-70932-z)
Supplement: Supplementary file 1 — Supplementary Legends. [file 41598_2020_70932_MOESM1_ESM.docx]

Figure S1. Distribution of differentially expressed transcripts/genes (DEGs) involved in biological processes, molecular functions and cellular components in USVL677-PMS at 8 dpi. (**A**) Gene ontology (GO) biological process and molecular function (https://www.blast2go.com/)^30^. (**B**) Comparative expression of up-regulated and down-regulated DEGs associated with molecular functions (MF) in USVL677-PMS at 8 dpi (**C**) Comparative expression of up-regulated and down-regulated DEGs associated with biological process (BP) in USVL677-PMS at 8 dpi (**D**) Venn graph representing the distribution of upregulated & downregulated DEGs in USVL677-PMS at 8 dpi. VENNTURE^51^ software version 1.1.0.2 was used to generate the venn graph.

Figure S2. KEGG enrichment analysis of (**A**) up-regulated and down-regulated DEGs in PM susceptible line (USVL677-PMS). The x-axis represents the most enriched pathways, the y-axis represents the number of DEGs. The red bars represent the up-regulated DEGs, and the blue bars indicate the down-regulated DEGs. (B) Up-regulated and down-regulated DEGs link to KEGG pathway “plant-pathogen interaction” USVL677-PMS at 8 dpi. The KEGG pathway analysis was done using the KOBAS 3 database <http://kobas.cbi.pku.edu.cn/kobas3>. The plant-pathogen interaction image was generated by the KEGG database, [www.kegg.jp/kegg/kegg1.html](http://www.kegg.jp/kegg/kegg1.html)^52^.

Figure S3. KEGG enrichment analysis of DEGs in (**A**) Multiple Disease Resistance Line (MDR) and (**B**) PM susceptible line (PMS). The x-axis represents the most enriched pathways, the y-axis represents the number of DEGs. The red bars represent the up-regulated DEGs, and the green bars indicate the down-regulated DEGs. (**C**) Resistance response in USVL531-MDR and USVL677-PMS. The KEGG pathway analysis was done using the KOBAS 3 database <http://kobas.cbi.pku.edu.cn/kobas3> and the plant-pathogen interaction image was generated by the KEGG database, [www.kegg.jp/kegg/kegg1.html](http://www.kegg.jp/kegg/kegg1.html)^52^.

Figure S4. Blast2go graph (biological process) showing the Gene Ontology (GO) classification of all the differentially expressed genes DEGs/ transcripts (2566 genes) were categorized into broader GO classes using the GO enrichment and GO gene classification tools available in the Cucurbit Genomics Database http://cucurbitgenomics.org/pwyenrich and Blast2GO server (<https://www.blast2go.com/)30>.

Figure S5. Blast2go graph (molecular function) showing the Gene Ontology (GO) classification of all the differentially expressed genes DEGs/ transcripts (2566 genes) during compatible and incompatible interaction. Blast2go software was used to generate the GO annotations (<https://www.blast2go.com/)30>.

Figure S6. Real time data on DEGs

Supplementary Tables

Table S1. List of DEGs in response to *P. xanthii*.

Table S2. 27 genes list involved in regulation of gene expression.

Table S3. KEGG Pathway_8dpi_PMS

Table S4. KEGG_Pathways_MDR vs PMS_alltimepoints

Table S5. List of NBS-LRR and MLO genes with expression change.

Table S6. 31 common genes list induced in defense signaling pathway in USVL531-MDR.

Table S7.1. List of upregulated DEGs in USVL531-MDR involved in biological process

Table S7.2. List of downregulated DEGs in USVL677-PMS involved in biological process

Table S8. Detailed predicted secondary structure sequence information of *ClaPMR2* (alpha helix, beta strand, coil and turn)

Table S9. KEGG_Pathways_MDR 0 h vs PMS_0 h

Table S10. Real time Primer lists
