# Supplementary material for: Elucidation of resistance signaling and identification of powdery mildew resistant mapping loci (ClaPMR2) during watermelon-Podosphaera xanthii interaction using RNA-Seq and whole-genome resequencing approach
Source: Sci Rep. 2020 Aug 20;10:14038. doi: 10.1038/s41598-020-70932-z (PMC7441409; doi:10.1038/s41598-020-70932-z)
Supplement: Supplementary file 2 — Supplementary Fig. S1. [file 41598_2020_70932_MOESM2_ESM.pptx]

## Slide 1
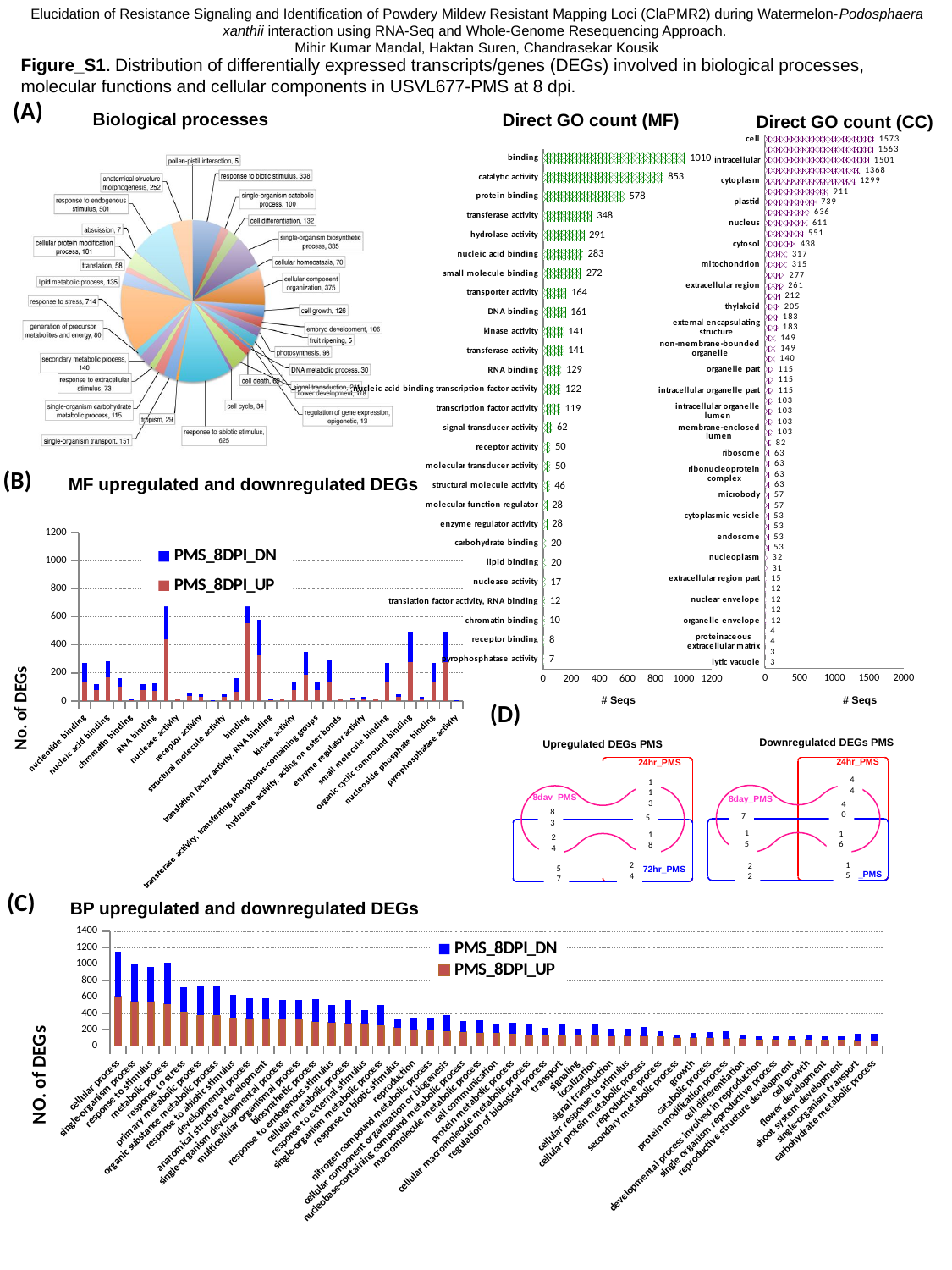

Elucidation of Resistance Signaling and Identification of Powdery Mildew Resistant Mapping Loci (ClaPMR2) during Watermelon-Podosphaera xanthii interaction using RNA-Seq and Whole-Genome Resequencing Approach.
Mihir Kumar Mandal, Haktan Suren, Chandrasekar Kousik
Figure_S1. Distribution of differentially expressed transcripts/genes (DEGs) involved in biological processes,
molecular functions and cellular components in USVL677-PMS at 8 dpi.
(A)
Biological processes
Direct GO count (MF)
Direct GO count (CC)
### Chart
| Category | |
|---|---|
| pyrophosphatase activity | 7.0 |
| receptor binding | 8.0 |
| chromatin binding | 10.0 |
| translation factor activity, RNA binding | 12.0 |
| nuclease activity | 17.0 |
| lipid binding | 20.0 |
| carbohydrate binding | 20.0 |
| enzyme regulator activity | 28.0 |
| molecular function regulator | 28.0 |
| structural molecule activity | 46.0 |
| molecular transducer activity | 50.0 |
| receptor activity | 50.0 |
| signal transducer activity | 62.0 |
| transcription factor activity | 119.0 |
| nucleic acid binding transcription factor activity | 122.0 |
| RNA binding | 129.0 |
| transferase activity | 141.0 |
| kinase activity | 141.0 |
| DNA binding | 161.0 |
| transporter activity | 164.0 |
| small molecule binding | 272.0 |
| nucleic acid binding | 283.0 |
| hydrolase activity | 291.0 |
| transferase activity | 348.0 |
| protein binding | 578.0 |
| catalytic activity | 853.0 |
| binding | 1010.0 |
### Chart
| Category | |
|---|---|
| lytic vacuole | 3.0 |
| lysosome | 3.0 |
| proteinaceous extracellular matrix | 4.0 |
| extracellular matrix | 4.0 |
| organelle envelope | 12.0 |
| extracellular space | 12.0 |
| nuclear envelope | 12.0 |
| envelope | 12.0 |
| extracellular region part | 15.0 |
| cytoskeleton | 31.0 |
| nucleoplasm | 32.0 |
| vesicle | 53.0 |
| endosome | 53.0 |
| intracellular vesicle | 53.0 |
| cytoplasmic vesicle | 53.0 |
| peroxisome | 57.0 |
| microbody | 57.0 |
| macromolecular complex | 63.0 |
| ribonucleoprotein complex | 63.0 |
| intracellular ribonucleoprotein complex | 63.0 |
| ribosome | 63.0 |
| nucleolus | 82.0 |
| membrane-enclosed lumen | 103.0 |
| organelle lumen | 103.0 |
| intracellular organelle lumen | 103.0 |
| nuclear lumen | 103.0 |
| intracellular organelle part | 115.0 |
| nuclear part | 115.0 |
| organelle part | 115.0 |
| endoplasmic reticulum | 140.0 |
| non-membrane-bounded organelle | 149.0 |
| intracellular non-membrane-bounded organelle | 149.0 |
| external encapsulating structure | 183.0 |
| cell wall | 183.0 |
| thylakoid | 205.0 |
| Golgi apparatus | 212.0 |
| extracellular region | 261.0 |
| vacuole | 277.0 |
| mitochondrion | 315.0 |
| endomembrane system | 317.0 |
| cytosol | 438.0 |
| plasma membrane | 551.0 |
| nucleus | 611.0 |
| cell periphery | 636.0 |
| plastid | 739.0 |
| membrane | 911.0 |
| cytoplasm | 1299.0 |
| intracellular organelle | 1368.0 |
| intracellular | 1501.0 |
| cell part | 1563.0 |
| cell | 1573.0 |
(B)
MF upregulated and downregulated DEGs
### Chart
| Category | PMS_8DPI_UP | PMS_8DPI_DN |
|---|---|---|
| nucleotide binding | 139.0 | 133.0 |
| nucleic acid binding transcription factor activity | 79.0 | 43.0 |
| nucleic acid binding | 168.0 | 115.0 |
| DNA binding | 103.0 | 58.0 |
| chromatin binding | 7.0 | 3.0 |
| transcription factor activity, sequence-specific DNA binding | 77.0 | 42.0 |
| RNA binding | 71.0 | 58.0 |
| catalytic activity | 441.0 | 412.0 |
| nuclease activity | 9.0 | 8.0 |
| signal transducer activity | 37.0 | 25.0 |
| receptor activity | 30.0 | 20.0 |
| receptor binding | 3.0 | 5.0 |
| structural molecule activity | 28.0 | 18.0 |
| transporter activity | 65.0 | 99.0 |
| binding | 557.0 | 453.0 |
| protein binding | 328.0 | 250.0 |
| translation factor activity, RNA binding | 7.0 | 5.0 |
| lipid binding | 13.0 | 7.0 |
| kinase activity | 77.0 | 64.0 |
| transferase activity | 184.0 | 164.0 |
| transferase activity, transferring phosphorus-containing groups | 77.0 | 64.0 |
| hydrolase activity | 135.0 | 156.0 |
| hydrolase activity, acting on ester bonds | 9.0 | 8.0 |
| oxygen binding | 11.0 | 11.0 |
| enzyme regulator activity | 11.0 | 17.0 |
| carbohydrate binding | 16.0 | 4.0 |
| small molecule binding | 139.0 | 133.0 |
| molecular transducer activity | 30.0 | 20.0 |
| organic cyclic compound binding | 277.0 | 218.0 |
| molecular function regulator | 11.0 | 17.0 |
| nucleoside phosphate binding | 139.0 | 133.0 |
| heterocyclic compound binding | 277.0 | 218.0 |
| pyrophosphatase activity | 0.0 | 6.0 |# Seqs
# Seqs
(D)
Downregulated DEGs PMS
Upregulated DEGs PMS
24hr_PMS
8day_PMS
72hr_PMS
24hr_PMS
44
113
8day_PMS
40
65
832
752
15
16
18
24
15
24
22
57
72hr_PMS
(C)
BP upregulated and downregulated DEGs
### Chart
| Category | PMS_8DPI_UP | PMS_8DPI_DN |
|---|---|---|
| cellular process | 602.0 | 550.0 |
| single-organism process | 543.0 | 460.0 |
| response to stimulus | 541.0 | 420.0 |
| metabolic process | 515.0 | 504.0 |
| response to stress | 414.0 | 300.0 |
| primary metabolic process | 373.0 | 352.0 |
| organic substance metabolic process | 373.0 | 353.0 |
| response to abiotic stimulus | 342.0 | 283.0 |
| developmental process | 340.0 | 243.0 |
| anatomical structure development | 337.0 | 242.0 |
| single-organism developmental process | 332.0 | 235.0 |
| multicellular organismal process | 329.0 | 232.0 |
| biosynthetic process | 295.0 | 281.0 |
| response to endogenous stimulus | 289.0 | 212.0 |
| cellular metabolic process | 279.0 | 282.0 |
| response to external stimulus | 278.0 | 163.0 |
| single-organism metabolic process | 250.0 | 254.0 |
| response to biotic stimulus | 226.0 | 112.0 |
| reproduction | 205.0 | 146.0 |
| nitrogen compound metabolic process | 189.0 | 155.0 |
| cellular component organization or biogenesis | 184.0 | 191.0 |
| nucleobase-containing compound metabolic process | 171.0 | 138.0 |
| macromolecule metabolic process | 162.0 | 154.0 |
| cell communication | 160.0 | 113.0 |
| protein metabolic process | 149.0 | 141.0 |
| cellular macromolecule metabolic process | 137.0 | 125.0 |
| regulation of biological process | 133.0 | 90.0 |
| transport | 126.0 | 137.0 |
| signaling | 126.0 | 87.0 |
| localization | 126.0 | 137.0 |
| signal transduction | 125.0 | 86.0 |
| cellular response to stimulus | 125.0 | 86.0 |
| cellular protein metabolic process | 123.0 | 115.0 |
| reproductive process | 115.0 | 68.0 |
| secondary metabolic process | 100.0 | 40.0 |
| growth | 100.0 | 63.0 |
| catabolic process | 97.0 | 74.0 |
| protein modification process | 94.0 | 87.0 |
| cell differentiation | 88.0 | 44.0 |
| developmental process involved in reproduction | 78.0 | 43.0 |
| single organism reproductive process | 78.0 | 43.0 |
| reproductive structure development | 78.0 | 43.0 |
| cell growth | 77.0 | 49.0 |
| flower development | 76.0 | 42.0 |
| shoot system development | 76.0 | 42.0 |
| single-organism transport | 67.0 | 84.0 |
| carbohydrate metabolic process | 65.0 | 86.0 |
