# Supplementary material for: Elucidation of resistance signaling and identification of powdery mildew resistant mapping loci (ClaPMR2) during watermelon-Podosphaera xanthii interaction using RNA-Seq and whole-genome resequencing approach
Source: Sci Rep. 2020 Aug 20;10:14038. doi: 10.1038/s41598-020-70932-z (PMC7441409; doi:10.1038/s41598-020-70932-z)
Supplement: Supplementary file 4 — Supplementary Fig. S3. [file 41598_2020_70932_MOESM4_ESM.pptx]

## Slide 1
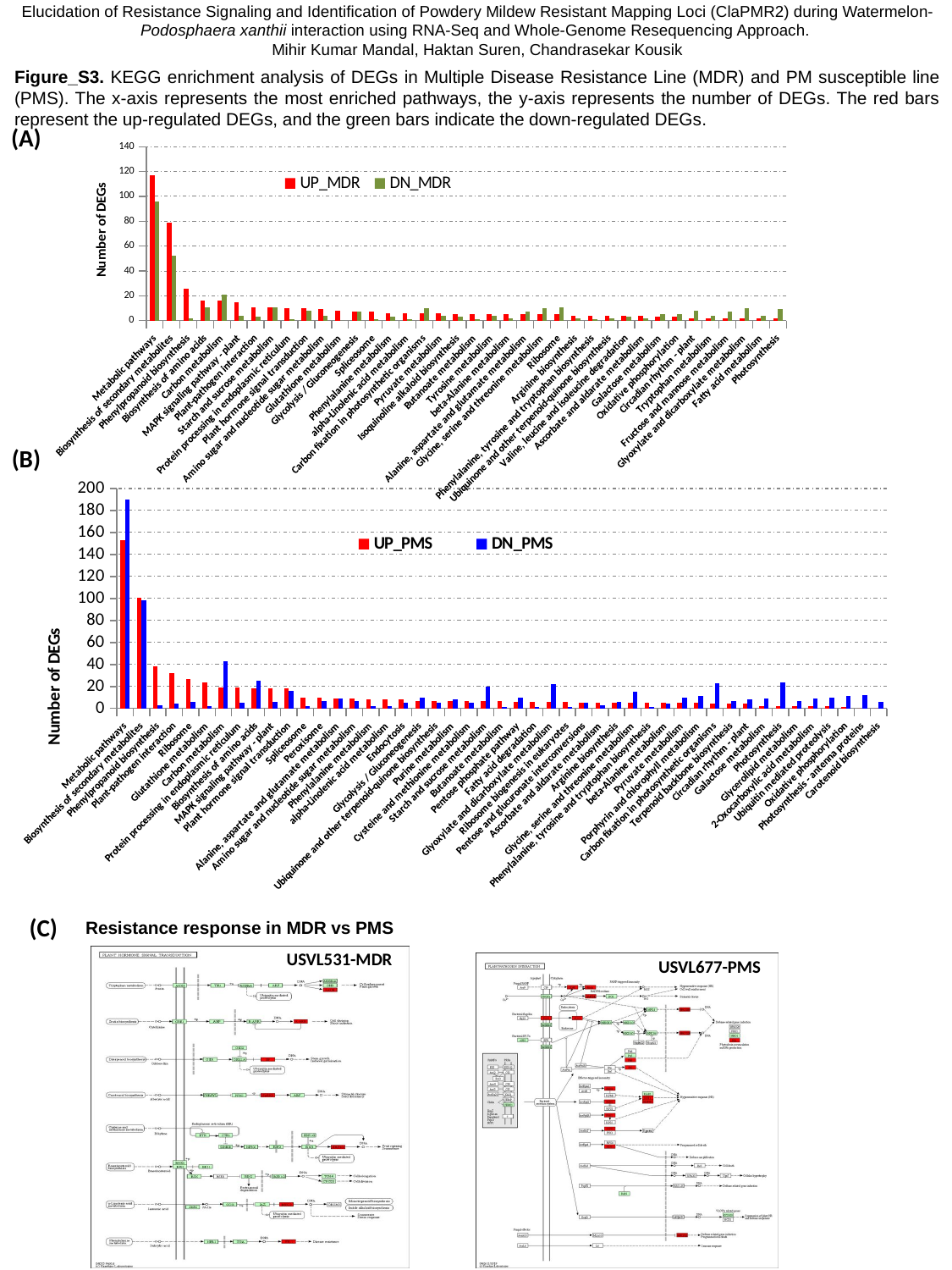

Elucidation of Resistance Signaling and Identification of Powdery Mildew Resistant Mapping Loci (ClaPMR2) during Watermelon-Podosphaera xanthii interaction using RNA-Seq and Whole-Genome Resequencing Approach.
Mihir Kumar Mandal, Haktan Suren, Chandrasekar Kousik
Figure_S3. KEGG enrichment analysis of DEGs in Multiple Disease Resistance Line (MDR) and PM susceptible line (PMS). The x-axis represents the most enriched pathways, the y-axis represents the number of DEGs. The red bars represent the up-regulated DEGs, and the green bars indicate the down-regulated DEGs.
(A)
### Chart
| Category | UP_MDR | DN_MDR |
|---|---|---|
| Metabolic pathways | 117.0 | 96.0 |
| Biosynthesis of secondary metabolites | 79.0 | 52.0 |
| Phenylpropanoid biosynthesis | 26.0 | 2.0 |
| Biosynthesis of amino acids | 16.0 | 11.0 |
| Carbon metabolism | 16.0 | 21.0 |
| MAPK signaling pathway - plant | 15.0 | 4.0 |
| Plant-pathogen interaction | 11.0 | 3.0 |
| Starch and sucrose metabolism | 11.0 | 11.0 |
| Protein processing in endoplasmic reticulum | 10.0 | 1.0 |
| Plant hormone signal transduction | 10.0 | 8.0 |
| Amino sugar and nucleotide sugar metabolism | 9.0 | 4.0 |
| Glutathione metabolism | 8.0 | 0.0 |
| Glycolysis / Gluconeogenesis | 7.0 | 7.0 |
| Spliceosome | 7.0 | 1.0 |
| Phenylalanine metabolism | 6.0 | 3.0 |
| alpha-Linolenic acid metabolism | 6.0 | 1.0 |
| Carbon fixation in photosynthetic organisms | 6.0 | 10.0 |
| Pyruvate metabolism | 6.0 | 4.0 |
| Isoquinoline alkaloid biosynthesis | 5.0 | 3.0 |
| Butanoate metabolism | 5.0 | 1.0 |
| Tyrosine metabolism | 5.0 | 4.0 |
| beta-Alanine metabolism | 5.0 | 2.0 |
| Alanine, aspartate and glutamate metabolism | 5.0 | 7.0 |
| Glycine, serine and threonine metabolism | 5.0 | 10.0 |
| Ribosome | 5.0 | 11.0 |
| Arginine biosynthesis | 4.0 | 2.0 |
| Phenylalanine, tyrosine and tryptophan biosynthesis | 4.0 | 1.0 |
| Ubiquinone and other terpenoid-quinone biosynthesis | 4.0 | 2.0 |
| Valine, leucine and isoleucine degradation | 4.0 | 3.0 |
| Ascorbate and aldarate metabolism | 4.0 | 2.0 |
| Galactose metabolism | 3.0 | 5.0 |
| Oxidative phosphorylation | 3.0 | 5.0 |
| Circadian rhythm - plant | 2.0 | 8.0 |
| Tryptophan metabolism | 2.0 | 4.0 |
| Fructose and mannose metabolism | 2.0 | 7.0 |
| Glyoxylate and dicarboxylate metabolism | 2.0 | 10.0 |
| Fatty acid metabolism | 2.0 | 4.0 |
| Photosynthesis | 2.0 | 9.0 |(B)
### Chart
| Category | UP_PMS | DN_PMS |
|---|---|---|
| Metabolic pathways | 153.0 | 190.0 |
| Biosynthesis of secondary metabolites | 101.0 | 98.0 |
| Phenylpropanoid biosynthesis | 38.0 | 3.0 |
| Plant-pathogen interaction | 32.0 | 4.0 |
| Ribosome | 27.0 | 6.0 |
| Glutathione metabolism | 24.0 | 2.0 |
| Carbon metabolism | 19.0 | 43.0 |
| Protein processing in endoplasmic reticulum | 19.0 | 5.0 |
| Biosynthesis of amino acids | 18.0 | 25.0 |
| MAPK signaling pathway - plant | 18.0 | 6.0 |
| Plant hormone signal transduction | 18.0 | 16.0 |
| Spliceosome | 10.0 | 2.0 |
| Peroxisome | 10.0 | 7.0 |
| Alanine, aspartate and glutamate metabolism | 9.0 | 9.0 |
| Amino sugar and nucleotide sugar metabolism | 9.0 | 7.0 |
| Phenylalanine metabolism | 8.0 | 2.0 |
| alpha-Linolenic acid metabolism | 8.0 | 2.0 |
| Endocytosis | 8.0 | 5.0 |
| Glycolysis / Gluconeogenesis | 7.0 | 10.0 |
| Ubiquinone and other terpenoid-quinone biosynthesis | 7.0 | 5.0 |
| Purine metabolism | 7.0 | 8.0 |
| Cysteine and methionine metabolism | 7.0 | 5.0 |
| Starch and sucrose metabolism | 7.0 | 20.0 |
| Butanoate metabolism | 7.0 | 1.0 |
| Pentose phosphate pathway | 6.0 | 10.0 |
| Fatty acid degradation | 6.0 | 1.0 |
| Glyoxylate and dicarboxylate metabolism | 6.0 | 22.0 |
| Ribosome biogenesis in eukaryotes | 6.0 | 1.0 |
| Pentose and glucuronate interconversions | 5.0 | 5.0 |
| Ascorbate and aldarate metabolism | 5.0 | 3.0 |
| Arginine biosynthesis | 5.0 | 6.0 |
| Glycine, serine and threonine metabolism | 5.0 | 15.0 |
| Phenylalanine, tyrosine and tryptophan biosynthesis | 5.0 | 1.0 |
| beta-Alanine metabolism | 5.0 | 4.0 |
| Pyruvate metabolism | 5.0 | 10.0 |
| Porphyrin and chlorophyll metabolism | 5.0 | 11.0 |
| Carbon fixation in photosynthetic organisms | 4.0 | 23.0 |
| Terpenoid backbone biosynthesis | 4.0 | 7.0 |
| Circadian rhythm - plant | 4.0 | 8.0 |
| Galactose metabolism | 2.0 | 9.0 |
| Photosynthesis | 2.0 | 24.0 |
| Glycerolipid metabolism | 2.0 | 7.0 |
| 2-Oxocarboxylic acid metabolism | 2.0 | 9.0 |
| Ubiquitin mediated proteolysis | 2.0 | 10.0 |
| Oxidative phosphorylation | 1.0 | 11.0 |
| Photosynthesis - antenna proteins | 0.0 | 12.0 |
| Carotenoid biosynthesis | 0.0 | 6.0 |(C)
Resistance response in MDR vs PMS
USVL531-MDR
USVL677-PMS
