# Supplementary material for: Elucidation of resistance signaling and identification of powdery mildew resistant mapping loci (ClaPMR2) during watermelon-Podosphaera xanthii interaction using RNA-Seq and whole-genome resequencing approach
Source: Sci Rep. 2020 Aug 20;10:14038. doi: 10.1038/s41598-020-70932-z (PMC7441409; doi:10.1038/s41598-020-70932-z)

Elucidation of Resistance Signaling and Identification of Powdery Mildew Resistant Mapping Loci (ClaPMR2) during Watermelon-*Podosphaera xanthii* interaction using RNA-Seq and Whole-Genome Resequencing Approach.

Mihir Kumar Mandal, Haktan Suren, Chandrasekar Kousik

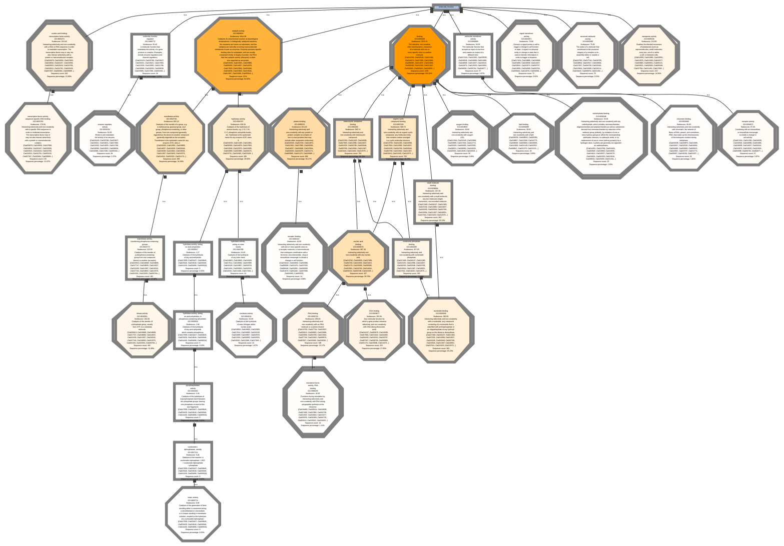

Supplement: Supplementary file 6 — Supplementary Fig. S5. [file 41598_2020_70932_MOESM6_ESM.pdf]
