# Supplementary material for: Elucidation of resistance signaling and identification of powdery mildew resistant mapping loci (ClaPMR2) during watermelon-Podosphaera xanthii interaction using RNA-Seq and whole-genome resequencing approach
Source: Sci Rep. 2020 Aug 20;10:14038. doi: 10.1038/s41598-020-70932-z (PMC7441409; doi:10.1038/s41598-020-70932-z)
Supplement: Supplementary file 7 — Supplementary Fig. S6. [file 41598_2020_70932_MOESM7_ESM.pptx]

## Slide 1
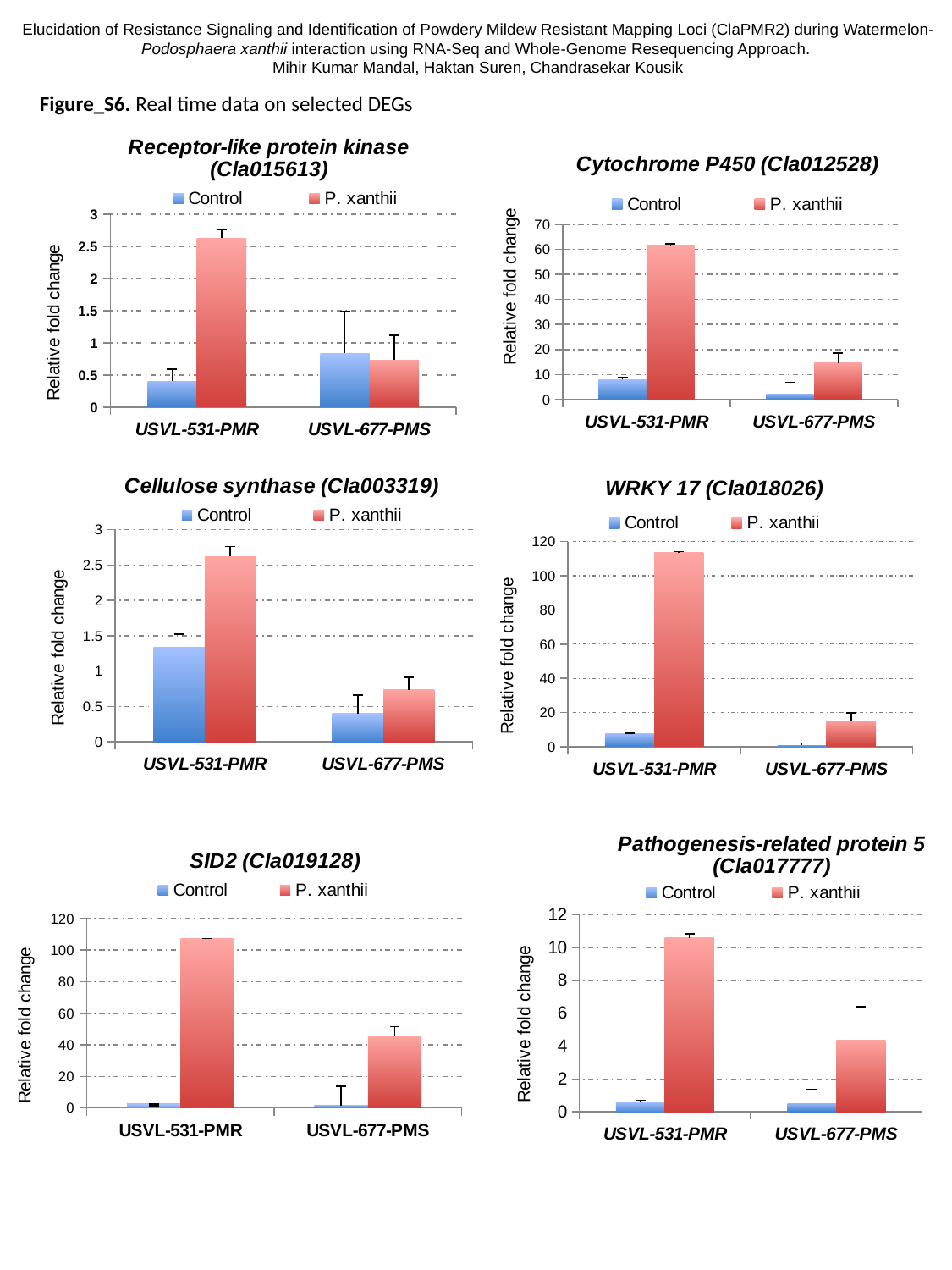

Elucidation of Resistance Signaling and Identification of Powdery Mildew Resistant Mapping Loci (ClaPMR2) during Watermelon-Podosphaera xanthii interaction using RNA-Seq and Whole-Genome Resequencing Approach.
Mihir Kumar Mandal, Haktan Suren, Chandrasekar Kousik
Figure_S6. Real time data on selected DEGs
### Chart: Receptor-like protein kinase (Cla015613)
| Category | Control | P. xanthii |
|---|---|---|
| USVL-531-PMR | 0.400480807930421 | 2.62437761277555 |
| USVL-677-PMS | 0.833504783887601 | 0.73349574314556 |
### Chart: Cytochrome P450 (Cla012528)
| Category | Control | P. xanthii |
|---|---|---|
| USVL-531-PMR | 7.987208222868871 | 61.52615499243651 |
| USVL-677-PMS | 2.08424452635614 | 14.53501046858485 |
### Chart: Cellulose synthase (Cla003319)
| Category | Control | P. xanthii |
|---|---|---|
| USVL-531-PMR | 1.33504783887602 | 2.62437761277555 |
| USVL-677-PMS | 0.400480807930421 | 0.73349574314556 |
### Chart: WRKY 17 (Cla018026)
| Category | Control | P. xanthii |
|---|---|---|
| USVL-531-PMR | 7.596811342246766 | 113.4722408544621 |
| USVL-677-PMS | 0.840260279862097 | 15.22211215054089 |
### Chart: Pathogenesis-related protein 5 (Cla017777)
| Category | Control | P. xanthii |
|---|---|---|
| USVL-531-PMR | 0.620025727558934 | 10.59381877825792 |
| USVL-677-PMS | 0.498240778942092 | 4.383402586482274 |
### Chart: SID2 (Cla019128)
| Category | Control | P. xanthii |
|---|---|---|
| USVL-531-PMR | 2.516396885063703 | 107.2311355046632 |
| USVL-677-PMS | 1.511069448687154 | 45.2323981907121 |
